# Large-scale perfused tissues via synthetic 3D soft microfluidics

**DOI:** 10.1038/s41467-022-35619-1

**Published:** 2023-01-12

**Authors:** Sergei Grebenyuk, Abdel Rahman Abdel Fattah, Manoj Kumar, Burak Toprakhisar, Gregorius Rustandi, Anja Vananroye, Idris Salmon, Catherine Verfaillie, Mark Grillo, Adrian Ranga

**Affiliations:** 1 Laboratory of Bioengineering and Morphogenesis, Biomechanics Section, Department of Mechanical Engineering, KU Leuven, Leuven, Belgium; 2 Stem Cell Institute Leuven and Department of Development and Regeneration, Faculty of Medicine, KU Leuven, Leuven, Belgium; 3 Laboratory of Soft Matter, Rheology and Technology, Department of Chemical Engineering, KU Leuven, Leuven, Belgium; 4 Grillo Consulting Inc., San Francisco, CA USA; 5 Leuven Brain Institute, KU Leuven, Leuven, Belgium; 6 Leuven Institute for Single Cell Omics, KU Leuven, Leuven, Belgium

**Keywords:** Stem-cell differentiation, Cell lineage, Biomaterials - cells, Lab-on-a-chip, Biomedical engineering

## Abstract

The vascularization of engineered tissues and organoids has remained a major unresolved challenge in regenerative medicine. While multiple approaches have been developed to vascularize in vitro tissues, it has thus far not been possible to generate sufficiently dense networks of small-scale vessels to perfuse large de novo tissues. Here, we achieve the perfusion of multi-mm^3^ tissue constructs by generating networks of synthetic capillary-scale 3D vessels. Our 3D soft microfluidic strategy is uniquely enabled by a 3D-printable 2-photon-polymerizable hydrogel formulation, which allows for precise microvessel printing at scales below the diffusion limit of living tissues. We demonstrate that these large-scale engineered tissues are viable, proliferative and exhibit complex morphogenesis during long-term in-vitro culture, while avoiding hypoxia and necrosis. We show by scRNAseq and immunohistochemistry that neural differentiation is significantly accelerated in perfused neural constructs. Additionally, we illustrate the versatility of this platform by demonstrating long-term perfusion of developing neural and liver tissue. This fully synthetic vascularization platform opens the door to the generation of human tissue models at unprecedented scale and complexity.

## Abstract (web summary)

Bioengineering live tissues has remained challenging due to limited nutrient exchange in the growing tissues. Here, the authors have developed micro-perfused 2-photon printing of 3D microfluidics, to engineer large-scale, viable and functional neural and hepatic 3D tissues.

## Introduction

Human engineered tissue and organoids are potentially transformational model systems, which could create dramatic efficiencies in the drug discovery process and function as key building blocks for regenerative medicine applications. In particular, larger-scale tissues have the possibility to recapitulate complex functional and organizational characteristics of their in vivo counterparts, and could therefore become a long-sought alternative to animal models^1–3^. However, the poorly defined structural organization, small size and slow maturation of these tissues have remained major limitations in engineering fully functional and reproducible organoids and tissues.

In vivo, the development of tissues is supported by a complex network of blood vessels which provide oxygen, nutrients and waste exchange and mediate paracrine interactions via growth and differentiation factors^4^. The size of the microvasculature is a critical parameter for local tissue perfusion: to maintain sufficient diffusion of oxygen, nutrients, and waste products most cells in vivo lie within 200 μm of a capillary. In the absence of vascular support, normal physiological conditions can be maintained only within this narrow range. Similar to the diffusion limits in normal tissue, the generation of solid tissue in vitro requires both vascularization and flow to maintain cell viability throughout the entire construct^1^. The lack of vascularization in engineered tissues therefore prevents oxygen and nutrient exchange, which is thought to be the main reason for the commonly observed development of a necrotic core within organoids once they reach a critical size, as well as for apoptosis within engineered tissues. These issues have been widely recognized, and various approaches have been reported in order to overcome them^5^.

The extrinsic induction of angiogenesis has been frequently used in the context of organoid vascularization. Vessel sprouts have been shown to infiltrate organoids maintained with endothelial cells in separate compartments of microfluidic culture devices^6,7^, or co-cultured with pre-established microvascular beds^8,9^. These results have suggested that the presence of a perfusable vasculature can enhance organoid growth^8^, confirming the importance of systemic cross-talk between vasculature and organoids in the developmental process. The resulting organoids have nonetheless been limited in size. Several approaches have been reported to generate functional organoid vascularization, including by grafting organoids into host animals^10,11^, overexpression of the transcription factor human ETS variant 2 (hETV2) in human embryonic stem cells^12^ or greatly enhancing pro-endothelial differentiation of endothelial progenitor cells in renal organoids by ambient flow^13^. With these approaches however, it is difficult to achieve a controlled flow rates as well as defined and reproducible topology of the micro-capillary network.

A number of studies have focused on creating artificial vessels through the use of templating approaches based on patterned layer-by-layer deposition of gelled material, in the form of thin filaments^14^, droplets^15–20^ or layer-by-layer polymerization by stereolithography^21–23^. Large tissue constructs have been generated by bioprinting of bioinks comprised of gels carrying different cell types^14,24,25^, with vascular templates generated by depositing endothelial cells interleaved with tissue-specific cells using filament extrusion^26–30^ or stereolithography^21,23^. While multiphoton lithography has been used to fabricate capillary-sized tubular fragments^31^ and vascular mimics^32^, these vessels have thus far not been successfully integrated in a complete perfusion platform. The dissolution of sacrificial networks to form lumenized vessels has been proposed as an alternative strategy, with resorbable gel filaments being created via stereolithography^33^, pre-polymer extrusion^34–40^ or molding^41^. A similar approach, referred to as the Kenzan method, uses an array of micro-needles as temporary structural supports^42–44^. Various types of tissues have been generated from pre-made spheroids by inserting needles into spheroids, which fuse into continuous tissue after several days, resulting in perfusable channels of 150–200 µm in diameter after removal of the needles. Alternatively, artificial vessels have also been formed by the direct removal of diverse hydrogel material such as silk fibroin and PEG hydrogels using laser photo-ablation^45–49^ including in the presence of cells^50–52^.

Despite the versatility of these vessel templating approaches, the minimal diameter of engineered vessels that have been integrated into a perfusion system thus far has been limited to 150 µm^35,41^. In parallel with strategies based on extrinsic angiogenesis in organ-on-chip implementations^53^, the size of generated tissues, particularly those requiring extensive blood flow (e.g. brain, liver, heart) has been limited to 400–500 µm in at least one dimension^54–58^. Because of their small size, engineered tissues which have been implemented thus far do not preserve a physiologically relevant signaling context within the tissue, nor do they develop to a level of complexity comparable to in vivo organs. Here we show through the use of 2-photon-mediated 3D microfluidics the fabrication of highly multiplexed synthetic vessels, enabling long-term perfusion of multi-millimeter scale engineered tissues.

## Results

### Photo-polymerizable non-swelling hydrogels enable 3D soft microfluidics

**Fig. 1:**
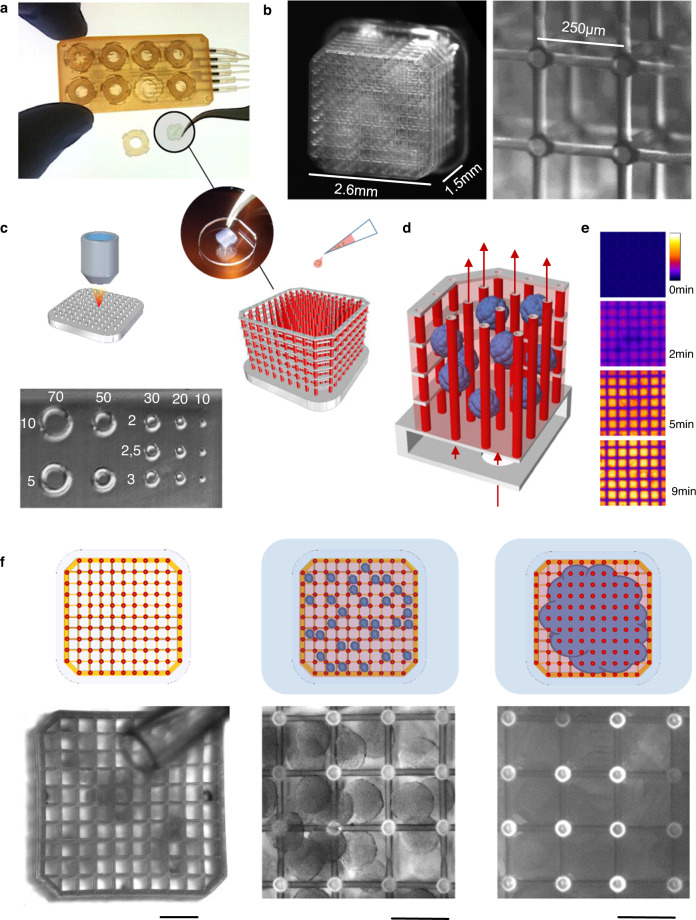
On-chip micro-vascularization enabled by soft microfluidics. **a** 3D printed tissue culture chip designed for eight multiplexed 3D soft microfluidic capillary grids. Inset: microfluidic capillary grid fabricated on a plastic baseplate and diagram of working principle. **b** Microfluidic capillary grid (left) and close up image of the same grid showing individual hydrogel capillaries (right). The manufacturing process generates capillary grids with about 90% success rate, as assessed over more than  one hundred produced devices. **c** Microvessel 3D printing of microfluidic grid using high-resolution 2-photon stereo-lithography with non-swelling photo-polymerizable hydrogel precursors enables reproduction of features as small as 10 µm. Printed examples demonstrate an array of cylinders of various outer diameters (top row) and wall thickness (columns) - units in µm. **d** CAD image of microfluidic grid (left) with capillaries shown in red and the structural components shown in gray. The edge of the structure makes up a “basket” that can be filled (right) with cell aggregates (spheroids) which merge and produce a solid tissue incorporating hydrogel capillaries. **e** Hydrogel capillaries are readily permeable. 25 µM of fluorescein, perfused through the microfluidic grid embedded in Matrigel, across the capillary walls and saturates the gel within several minutes. Color scale represents the range of fluorescein concentrations from 0 to 25 µM. **f** Process of tissue generation. Photograph of an empty capillary grid with 200 µl pipette tip visible above the grid (left, scale bar 500 µm), close up image of the grid seeded with hPSC spheroids (middle, scale bar 250 µm) manually dispensed from the pipette shown on the left image, image of the spheroids (right, scale bar 250 µm) fused into a solid tissue after 24–36 h of culturing on chip. The top row schematically represents the same process, where spheroids and the resulting tissue are shown in blue.

In order to vascularize tissue at large scale, we hypothesized that a microfluidic approach which could bridge the capillary to tissue scale would be necessary to enable the perfusion of thick three-dimensional tissue constructs. We therefore designed a dense, regularly spaced capillary network whose tubular walls were made of a hydrogel allowing diffusion (Fig. 1a). Tissues growing within this soft grid-like hydrated network would interface with an external perfusion pumping system, circulating cell culture medium throughout the volume of the tissue (Fig. 1a and Supplementary Fig. 1). We developed an approach whereby the perfusable grid would be printed directly on a hard plastic base (Fig. 1a), thereby forming a tight seal. This base, which contains perfusion holes, would then be incorporated into a perfusion chip linked to a peristaltic pump circulating cell culture medium (Supplementary Fig. 1).

An important requirement of this platform was the need to have capillary-like tubing at scales of a few µm in diameter and thickness, while perfusing across a large, multi-mm^3^ three-dimensional space. The geometrical complexity of the design, properties of the biopolymer and fabrication scale ranging from 10 µm to 2000µm featured by our design made two-photon laser scanning photo-polymerization the ideal technology for this purpose. Initial 3D prints with the 2-photon Nanoscribe printer using commonly used photopolymerizable materials, including gelatin and PEG diacrylate, resulted in significant swelling of the material upon polymerization and hydration, which disrupted the seal between the soft microfluidic grid and the rigid plastic plate, and generated mismatched tubular segments (Supplementary Fig. 2).

To overcome this post-printing distortion, we developed a custom formulated hydrophilic photo-polymer based on polyethylene glycol diacrylate (PEGDA). While PEGDA exhibits cell-repelling surface properties, the polymer surface could be rendered cell-binding by the addition of the photocrosslinker pentaerythritol triacrylate (PETA)^59^. We reasoned that the addition of a significant amount of PETA as a crosslinker would increase the density  of the polymer, and balanced this with the addition of an inert “filler” component (Triton-X 100) to retain sufficient porosity to enable rapid diffusion. Rheometry measurements on the water-equilibrated photo-polymer indicated a storage modulus, G’ of 250 kPa, and a loss modulus G'' value an order of magnitude lower, with water content at 34% (Supplementary Fig. 2).

The combination of 2-photon printing with this non-swelling hydrogel material allowed printing of a variety of microfluidic grids, ranging in size from 1.2 × 1.2 × 1.2 mm up to 6.5 × 6.5 x 5 mm (Fig. 1b and Supplementary Fig. 3) with vessel diameters from 10 µm to >70 µm and vessel wall thickness from 2 µm to 10 µm (Fig. 1c). Importantly, the printing with our newly formulated resin resulted in a 1:1 fidelity between the generated CAD geometry and the printed parts, thereby ensuring no distortion and a tight seal (Supplementary Fig. 2). The standard size used for most of the subsequent experiments was 2.6 mm × 2.6 mm × 1.5 mm, with an inter-vessel distance of 250 µm (Fig. 1b). These grids could be incorporated into a multiplexed perfusion chip allowing up to 8 grids to be perfused simultaneously (Fig. 1a). Single cells or organoids smaller than the 250 µm inter-capillary distance, previously mixed within a liquid hydrogel (eg. Matrigel) precursor solution, could be seeded into the platform, yielding a “gel-in-gel” 3-dimensional construct (Fig. 1d). Vessel permeability to water-soluble molecules within the grids overlayed with Matrigel was verified using fluorescein, with diffusion throughout the three-dimensional space seen in <10 min (Fig. 1e).

To perform a biological proof of concept experiment, we generated hundreds of organoids of <200 µm diameter by aggregating human pluripotent stem cells (hPSCs) in microwells in pluripotency medium over 24 h. We collected these aggregates in cold liquid Matrgiel, which were then pipetted into the grids. Initial seeding demonstrated that these aggregates filled the grids and, over a period of 8 days of growth and neural differentiation, merged and filled the whole volume (Fig. 1f and Supplementary Fig. 4).

### scRNAseq reveals changes in differentiation, hypoxia, cell cycle regulation and differentiation upon perfusion

**Fig. 2:**
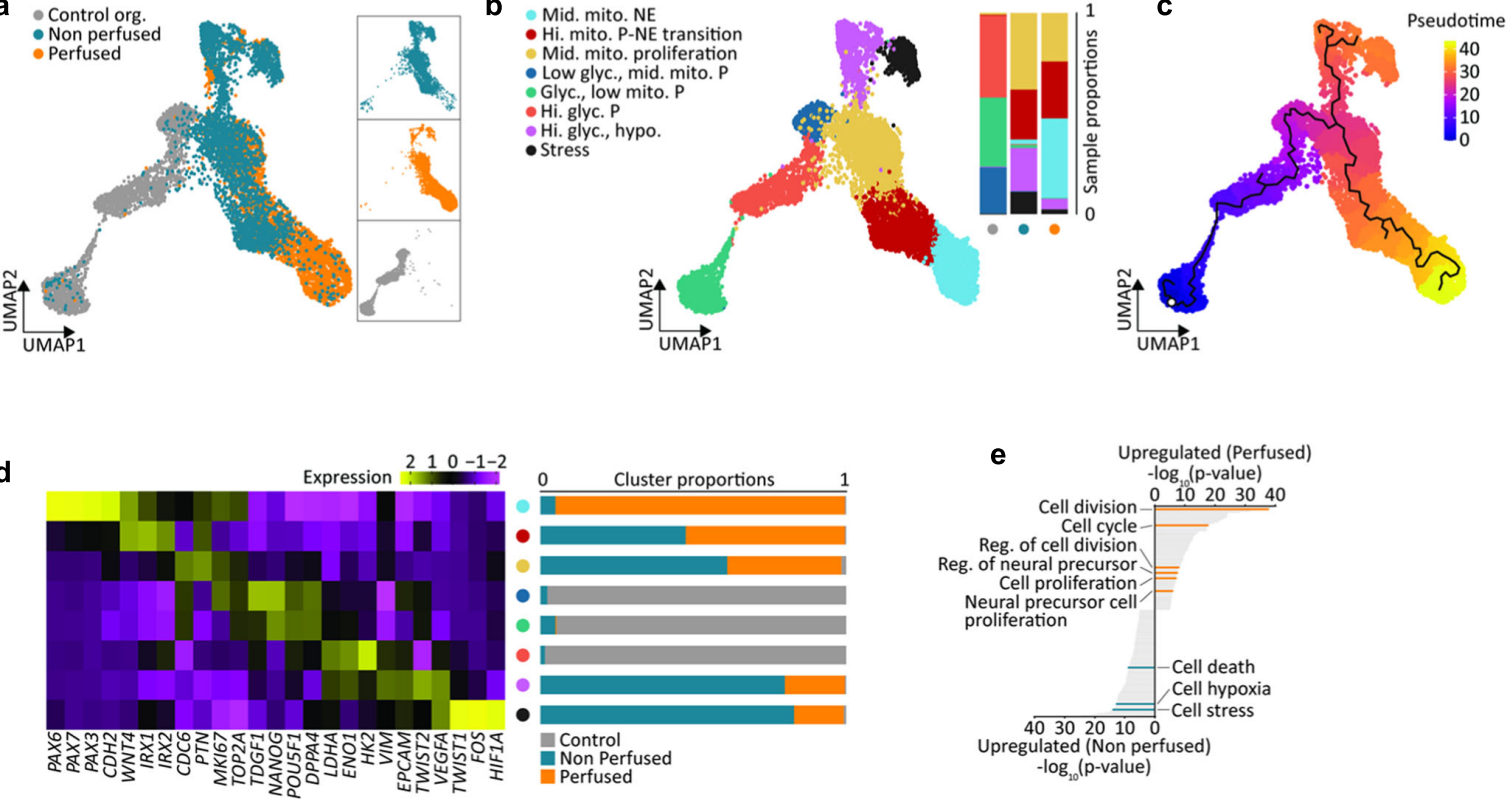
Transcriptomic changes upon perfusion. **a** Combined dataset UMAP (control, non-perfused and perfused samples). **b** Combined dataset UMAP with neuroepithelial cells (NE), pluripotent-neuroepithelial transitioning cells (P-NE), proliferating cells with medium mitochondrial (mito.) content, pluripotent cells (P) with low glycolysis (glyc.) and medium mitochondrial content, glycolytic pluripotent cells with low mitochondrial content, highly glycolytic pluripotent cells, a highly glycolytic hypoxic (hypo.) identity and a stressed cluster. **c** Pseudotime trajectory on combined dataset UMAP. **d** Cluster-specific expressions of selected marker genes. Color bar represents gene expression values, normalized and centered. Sample fractions for each identified cluster. **e** GO enrichment analysis for key processes upregulated in perfused and non-perfused samples.

To assess how perfusion affected cellular processes and differentiation in large-scale in-vitro tissue, we dissociated cells from tissue constructs in a perfused and a non-perfused grid, as well as from organoids in conventional suspension culture after the 8-day culture period, and performed single-cell RNA sequencing. Graph-based clustering and Uniform Manifold Approximation and Projection (UMAP) dimensionality reduction technique on the 8625 total cells retained after QC revealed significant transcriptomic differences between the tissue constructs (perfused and non-perfused) and the control organoids, as evidenced by largely separated clustering of these cell populations (Fig. 2a). Correlation analysis using the 100 most differentially expressed marker genes revealed the most difference between perfused tissue and conventional organoid culture, with non-perfused tissue sharing gene expression profiles with the other two conditions (Supplementary Fig. 5). Differential gene expression analysis was then used to annotate eight clusters, which were largely differentiated by fate, as well as by metabolic, hypoxia and cell cycle regulation (Fig. 2b, d, Supplementary Figs. 6, 7). Cells from control organoids were found in clusters with low, medium and high glycolytic processes marked by a pluripotent identity, including highly expressed markers such as *NANOG* and *OCT4* (*POUF5F1*). Both perfused and non-perfused tissues expressed varying degrees of hypoxia, proliferation, mitochondrial gene expression and neuroepithelial markers. The non-perfused tissue made up the largest proportion of the hypoxic and stressed clusters (*HIF1α*, *FOS*) while the perfused tissue represented the majority of the neuroepithelial cluster (*PAX6*).

In particular, control organoids had lower expression of mitochondrial genes (Supplementary Figs. 7, 8) compared to the tissue constructs. This is in line with previous reports of lower mitochondrial activity in human embryonic stem cells, which increases upon differentiation to fit the energy needs of resultant cell identities^60^. Indeed, differentiation towards the neuroepithelial fates from pluripotent cells was characterized by an increase in mitochondrial activity, and a simultaneous decrease in glycolysis (Supplementary Fig. 8). These results suggest a metabolic switch from anaerobic glycolysis to oxidative phosphorylation in the tissue constructs, as has been reported to occur during cell differentiation after exit from pluripotency^61,62^. Such transitions, which were additionally evidenced in pseudotime analysis (Fig. 2c) distinguished the control organoids from the tissue constructs in the microfluidic grids, and further characterized differences between non-perfused and perfused tissues.

The perfused tissue was also characterized by the expression of the neuroepithelial markers *PAX6*, *PAX7*, *PAX3*, and *CDH2*, while sharing *IRX1* and *IRX2* markers with non-perfused tissue (Fig. 2d). Moreover, while non-perfused and perfused tissues represented similar proportions of the pluripotent proliferating cluster, the perfused tissue expressed the highest proliferation markers such as *CDC6* (Supplementary Fig. 7). Additionally, the high expression of proliferation markers such *MKi67* and *TOP2A* in the neuroepithelial cluster suggested that perfused tissue retained proliferative capacity after neuroepithelial differentiation, while maintaining minimal hypoxic/stress response markers *VEGF*, *FOS*, *HIF1α* (Supplementary Fig. 8). In contrast, non-perfused tissue was distinguished by significant pluripotency (*NANOG*, *POU5F1*/*OCT-4*) (Fig. 2d) and hypoxia/stress markers (Fig. 2d, Supplementary Fig. 8). The control organoids, on the other hand, were mostly represented by pluripotent and hypoxic cells with complete absence of neuroepithelial identity (Fig. 2d and Supplementary Fig. 8), likely due to the short time in neural differentiation conditions.

To investigate transcriptional changes and associated cellular processes upon perfusion in a systematic manner, we performed differential gene expression analysis between perfused and non-perfused tissue constructs followed by Gene Ontology (GO) enrichment analysis (Fig. 2e). This analysis confirmed up-regulation of processes related to cell division and proliferation in the perfused sample, as well as regulation of neural precursors and neural precursor cell proliferation. In contrast, cell stress, hypoxia and cellular death processes were upregulated in non-perfused samples. Taken together our analysis of the transcriptomic data suggests that perfusion of large tissue constructs dramatically decreases apoptosis and hypoxia, and accelerates the process of neural differentiation.

### Perfusion rescues hypoxia and necrotic core in large tissue constructs

**Fig. 3:**
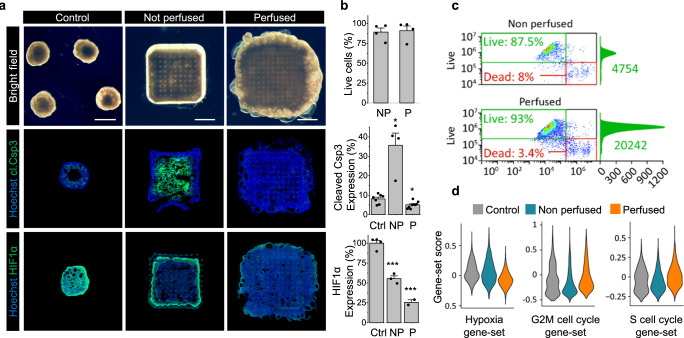
Changes in proliferation mediated by hypoxia. **a** Representative samples demonstrating differences in proliferation and viability between standard organoid culture (left column) and the tissue constructs without (middle column) and with perfusion (right column). Top row: bright field images of organoids, non-perfused and perfused constructs. Middle row: immunofluorescent images of apoptotic marker cleaved Caspase 3 (green) expressed in the three conditions. Bottom row: immunofluorescent images of hypoxia marker HIF1α (green). Hoechst staining of nuclei shown in blue. The images represent transverse cross-sections of the tissue constructs. The statistics on the biological replicates is given in **b**. Scale bar 1 mm. **b** Top: average proportion of the number of live cells in perfused and non-perfused constructs quantified by flow cytometry (90.9 ± 5.1% in perfused vs 89.2 ± 4.4% in non-perfused tissue, n.s., *n* = 4). Middle: average expression of cleaved Caspase 3 in control organoids (8 ± 1% area, *n* = 7), non-perfused (35.7 ± 5%, p_Control_ = 0.02, *n* = 4) and perfused tissue constructs (5.1 ± 1% of total area, p_Control_ = 0.02, *n* = 9). Bottom: average expression of hypoxia marker HIF1α in control organoids (100 ± 3%, *n* = 4), non-perfused (55 ± 3%, p_control_ = 0.0003, *n* = 3) and perfused constructs (26 ± 2%, p_control_ = 0.0004, *n* = 2). Control organoids, non-perfused and perfused constructs are denoted as Ctrl, NP and P respectively, data are derived from independent experiments and are represented as mean ± SEM, asterisks (*) denote statistical significance between control organoids and the tissue constructs (unpaired two-tailed Student’s *t*-test, 95% confidence interval). **c** Representative flow cytometry data, demonstrating the difference in total number of cells between perfused and non-perfused constructs. **d** Distribution of gene expression levels across the single-cell population for hypoxia (left), G2M cell cycle (middle) and S cell cycle (right) associated gene sets.

To further investigate how perfusion modulates the spatial distribution of hypoxia and apoptosis, we imaged the constructs in bright field microscopy and sectioned whole samples transversely, perpendicular to the direction of grid perfusion, followed by immunohistochemistry (Fig. 3a and associated quantification in Fig. 3b). Control organoids demonstrated a characteristic dark dense tissue core with occasional lighter void-like structures, surrounded by more translucent peripheral tissue. In non-perfused samples, tissue growth was restricted to the internal volume of the microfluidic grid, with generally dense central tissue interspersed with patchy lighter areas. Strikingly, perfused tissue covered the entire volume of the grid in a uniformly dense manner, with bulging epithelial outgrowths characteristic of cerebral organoids at the periphery. These observations suggest that cell proliferation was much higher in the perfused grids, compared to the non-perfused samples. To confirm this observation quantitatively, we dissociated the tissue constructs into single cells, stained with calcein-AM and ethidium homodimer to label live and dead cells respectively, followed by quantitative flow cytometry (Fig. 3b, c). Our analysis revealed a 5-fold difference in total cell number in the perfused tissue constructs over the non-perfused ones, while the proportion of live cells was similar (90.9 ± 5.1% in perfused vs 89.2 ± 4.4% in non-perfused tissue) (Fig. 3a, b).

To determine whether these changes in viability and proliferation were due to apoptosis, we stained sections of the grids for cleaved Caspase 3, an active form of the Caspase-3 enzyme responsible for the degradation of multiple cellular proteins and ultimately for cell fragmentation into apoptotic bodies. Upon sectioning, the control organoids were empty in the center, suggesting, as has previously been reported^63^, the loss of apoptotic cells during the sectioning process. Closer to this inner core, signs of apoptosis were evident (8 ± 1% of total area), (Fig. 3a, b), consistent with the large size of these organoids. Tissue in the non-perfused grids exhibited a clear inner core of apoptotic cellular fragments (35.7 ± 5% of total section area) (Fig. 3a) along with empty regions completely lacking cells, with some analogous features to control organoids. Importantly, nearly the entire perfused tissue did not show signs of cleaved Caspase 3 (5.1 ± 1% of total area), indicating that perfusion successfully prevented cell apoptosis throughout the course of differentiation.

To verify whether hypoxia could be involved in initiating the observed apoptosis in the non-perfused samples, we next stained for HIF1α, a heterodimer protein complex playing a key role in oxygen homeostasis^64,65^. The rapid buildup of HIF1α in low-oxygen conditions is known to trigger a hypoxic response ultimately leading to apoptosis^66,67^. In line with scRNA analysis data (Fig. 3d), high HIF1α expression levels were detected in the control organoids, as well as in the non-perfused samples (55 ± 3% of mean fluorescent intensity in organoid control). Conversely, low levels of HIF1α expression were observed in the perfused samples (26 ± 2% of organoid control) (Fig. 3a), associated with a higher proportion of cycling G2, M and S phase cells (Fig. 3d).

The patterns of HIF1α and cleaved Caspase 3 expression in non-perfused tissue sections therefore suggest that in these samples, cells in the center of the construct were in a transient hypoxic state ultimately leading to apoptotic cell death. This transition from hypoxic to apoptotic cell state was prevented in superficial layers of the non-perfused tissue where oxygen supply by passive diffusion was still sufficient to maintain cellular metabolism, with the accumulation of apoptotic bodies and cellular debris preserving the cleaved Caspase 3 expression in the bulk of the non-perfused tissue (Supplementary Fig. 9). Conversely, this phenomenon was completely absent in the perfused samples, clearly confirming that thick tissues at multi-mm^3^ scale could be grown with high viability, and with little to no apoptosis or hypoxia within this platform.

### Accelerated neural differentiation in perfused tissue constructs

**Fig. 4:**
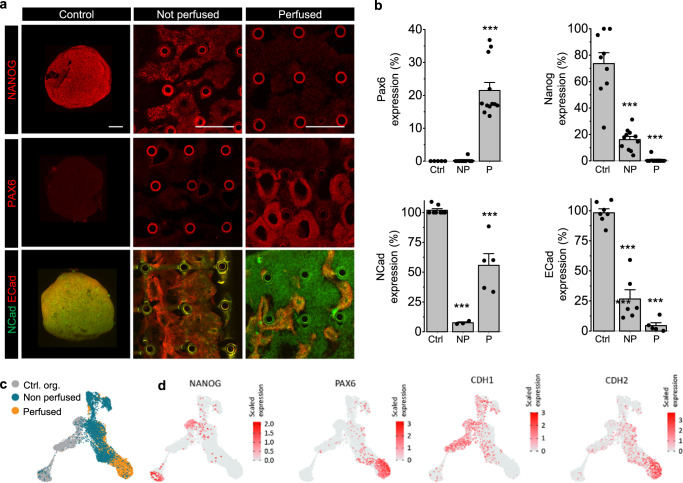
Rapid neural differentiation in perfused systems. **a** Representative experimental results demonstrating the result of 2 days of neuronal induction in control organoids (left column), non-perfused (middle column) and perfused (right column) tissue constructs. Top, middle and bottom row: immunofluorescent images of stem cell marker NANOG, early neural marker PAX6 and cell adhesion proteins N-Cadherin (green) and E-Cadherin (red). The images represent transverse cross-sections of the tissue constructs. In most cases, the cross-sections of capillaries are visible as circular structures. Statistics on biological replicates are given in **b**. **b** Average expression of PAX6 (Ctrl: Not detected, *n* = 5; NP: 0.2%±0.2%, p_Control_ = 0.35(n.s.), *n* = 9; P: 21.5 ± 2%, p_Control_ = 0.00002, *n* = 12), NANOG (Ctrl: 73.6 ± 8%, *n* = 9; NP: 16.1 ± 2%, p_Control_ = 0.00007, *n* = 11; P: not detected, *n* = 10), NCad (CDH2) (Ctrl: 100 ± 1%, *n* = 8; NP: 7.6 ± 1%, p_Control_ = 4e-13, *n* = 3; *P*: 55.7 ± 9%, p_Control_ = 0.009, *n* = 5) and ECad (CDH1) (Ctrl: 99 ± 2%, *n* = 12; NP: 26.6 ± 7%, p_Control_ = 0.0001, *n* = 6; *P*: 4.5 ± 2%, p_Control_ = 2e-10, *n* = 5) markers in control organoids (Ctrl), non-perfused (NP) and perfused (P) constructs. Data are derived from independent experiments and are represented as mean ± SEM, asterisks (*) denote statistical significance between control organoids and tissue constructs (unpaired two-tailed Student’s *t*-test, 95% confidence interval). **c** UMAP plot of the combined dataset showing the localization of cells from control organoids, non-perfused and perfused constructs in the UMAP space. Color bars represent scaled level of expression of the corresponding genes. **d** UMAP plot of the combined dataset highlighting locations of PAX6, NANOG, NCad (CDH2) and ECad (CDH1) expressing cells in the UMAP space. Scale bar: 250 µm.

Our scRNAseq data suggested that perfusion not only improved proliferation, prevented apoptosis and hypoxia, but could also direct fate specification. To confirm these findings, we analyzed specific markers of pluripotency and neural differentiation by immunohistochemistry (Fig. 4a and associated image quantification in Fig. 4b). NANOG, a canonical marker of pluripotency was abundantly expressed in organoids (73.6 ± 8% of cells) and significantly expressed in non-perfused constructs (16.1 ± 2%) at the protein level, but was completely missing in perfused samples. Conversely, PAX6, the earliest marker of neural differentiation was clearly evidenced in the perfused samples (21.5 ± 2%), but not in organoid controls and non-perfused samples. These results were in line with the scRNAseq data, which indicated that *NANOG*-expressing cells were present in the control and non-perfused samples, while *PAX6* was largely expressed in the perfused sample (Fig. 4c). The transition of stem cells from pluripotency to neural identity is regulated by a loss of cellular expression of E-CADHERIN (CDH1) and a gain of N-CADHERIN (CDH2) expression which drives neural differentiation by inhibiting FGF-mediated pathways^68^. In order to assess whether perfusion enhanced this transition, we stained for E-CADHERIN to evidence the epithelial state associated with pluripotency, and for N-CADHERIN to confirm the transition to early neuro-epithelial identity. Perfused samples were indeed largely N-CADHERIN positive (55.7 ± 9% vs 7.6 ± 1% in non-perfused samples), while more cells in the non-perfused samples maintained E-CADHERIN + identity (26.6 ± 7% vs 4.5 ± 2% in perfused samples). These results were confirmed by the scRNA data (Fig. 4c), indicating the predominant expression of N-CADHERIN in cells from perfused tissue, and E-CADHERIN in non-perfused and control tissue. Taken together, these results demonstrate that perfusion rapidly accelerates the transition from pluripotency to early neuroepithelial identity, while cells which lack perfusion remain in a state of pluripotency.

### Long term perfusion of cerebral organoids

**Fig. 5:**
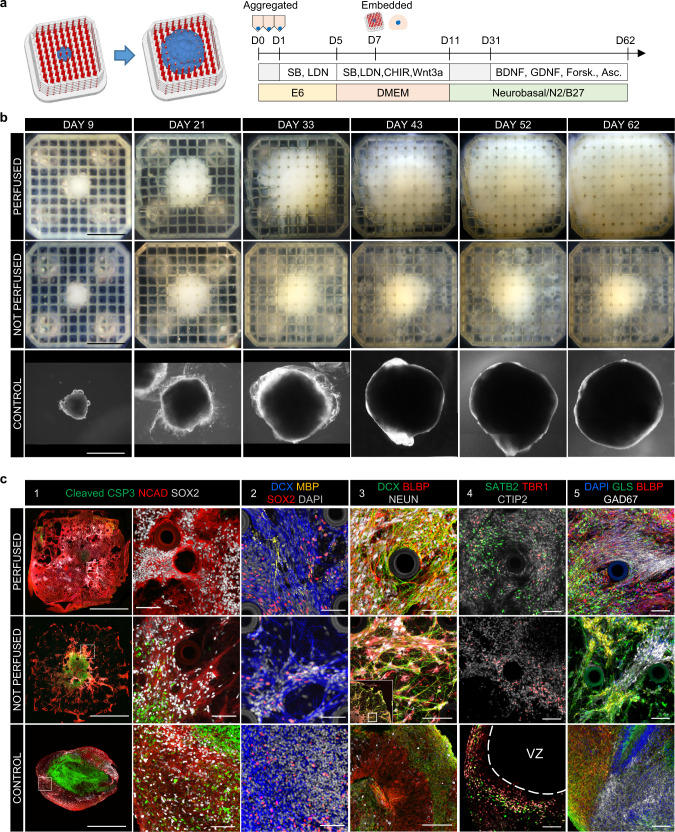
Long term perfused cortical organoids. **a** Schematic representation of the experiment and differentiation protocol. Single hPSC cells were aggregated in E6 medium for 24 h, then a directed multistep neuronal differentiation was conducted for 62 days. **b** Bright field images of organoids in perfused (top row), non-perfused (middle row) grids and control organoids (bottom row) over the culture period. Scale bar 1 mm. **c** Immunofluorescent images of transverse sections of the three sample types. **c1** Apoptotic marker cleaved Caspase 3 (green) indicates apoptotic cores in control organoids and non-perfused organoids, while healthy tissue shows neuronal identity (N-Cadherin, red, SOX2, white); non-perfused organoids have a characteristic mostly apoptotic dense core surrounded by sparse radially aligned cellular processes. Scale bars 1 mm and 70 µm on main and zoomed images respectively. **c2** perfused organoids exclusively express myelin basic protein (MBP, yellow), marker of mature oligodendrocytes, while all three conditions express immature, migratory cortical neuron marker Doublecortin (DCX, blue). Scale bar 70 µm. **c3** Staining for radial glia (BLBP (red) and DCX (green)) demonstrate a neuropil-like structure in perfused organoids formed by dense and intertwined networks of radial glia and neuronal processes, including a populations of mature neurons (NeuN, white), while in control organoids the cellular arborization is almost absent (also Supplementary Fig. 9b, c). Scale bar 70 µm. **c4** SATB2 (red), marker of upper cortical layers and TBR1(red) and CTIP2 (gray) markers of deeper layers are expressed throughout the perfused organoids and within the non-apoptotic regions of non-perfused organoids, while in control organoids, these markers are localized in the proximity of the ventricle-like structures. Scale bar 70 µm. **c5** Populations of glutamatergic (Glutaminase, green) and GABAergic (GAD67, white) neurons are expressed in all three conditions; while in perfused organoids a networked population is suggested by extended processes, in control organoids these neurons remain mainly segregated (also Supplementary Fig. 9d). Scale bar 70 µm. Representative images on (**b**) and (**c**) were acquired in three independent experiments; all experiments produced similar results.

We next sought to test the suitability of the platform for maintaining conventional single organoids over a longer differentiation period. In order to test this possibility we performed long term guided neuronal differentiation, based on an established cerebral organoid differentiation protocol^69^. In this set of experiments, single neural spheroids pre-differentiated for 5 days were embedded in Matrigel within perfused and non-perfused grids (Fig. 5a), while a third group of spheroids was embedded in Matrigel drops and cultured in suspension (control organoids).

During 2 months of differentiation, control organoids and organoids in perfused grids (perfused organoids) continuously grew to reach 2–2.5 mm in size by day 62 (Fig. 5b). Organoids in non-perfused grids (non-perfused organoids) formed a low-density fibrous tissue with a denser core of around 1 mm. We observed expression of cleaved Caspase 3 (cl.Csp3) in the cores of control and non-perfused organoids, indicating apoptosis in these areas (Fig. 5c). Perfused organoids exhibited minimal signs of apoptosis throughout the tissue construct, while in the control organoids a non-apoptotic (cl.Csp3-) area was observed in a 300–400 µm ring along the outer surface, similar to other reports^70,71^, and in the non-perfused organoids as a much thinner ring in the range of up to 200 µm. Perfused organoids developed multiple small cavities lined with N-Cad+ cells (Fig. 5c). We also found signs of myelination in perfused organoids, expressing myelin basic protein (MBP), a mature oligodendrocyte marker, which was not present in control or non-perfused organoids (Fig. 5c). Radial glial cells (RGs) play a key two-fold role in vertebrate brain development^72,73^. Serving as progenitor cells for neurons, astrocytes and oligodendrocytes, they also form an ordered physical scaffold constituted of long radial shafts that guide migration of newborn neurons in the developing cortical plate. We hypothesized that the observed difference in cytoarchitecture between control organoids and organoids in grids could be explained by a difference in the expression of radial glia cell network in the organoids. In perfused organoids, brain lipid binding protein (BLBP), a marker for developing radial glia, and Doublecortin (DCX), a marker of developing and migrating neurons, were expressed in large areas with extensive, dense and intertwined networks of BLBP + and DCX + cellular processes interspersed by highly scattered NeuN+ neurons. Remarkably, the processes of the two networks were frequently aligned and partially overlapped, suggesting a functional role of these RG cells in neuronal guidance (Fig. 5e, Supplementary Fig. 10a). RG processes in perfused organoids encircled the vessels, forming clear concentric patterns, while in other locations they aligned in a parallel pattern (Supplementary Fig. 10b). Non-perfused organoids developed sparse but extended BLBP + /DCX + bundles. In contrast, despite high expression of BLBP + and DCX + cells in control organoids, arborization of these cells was absent (Fig. 5e, Supplementary Fig. 10a) accompanied by only a sporadic expression of NeuN+ neurons in all organoids tested.

Human corticogenesis is characterized by the establishment of six specialized cortical layers expressing different neuronal subtype markers^74^. Around gestational week 8, approximately corresponding to the age our organoids, most cortical neurons in vivo, while presenting some initial layering, co-express markers of deep and upper neuronal layers, with expression of different cortical markers seen in largely overlapping cell populations^75,76^. In control organoids we detected extensive and overlapping populations of TBR1 + and CTIP2 + cells (layers 4, 5, 6), most of which were co-expressed, as well as a layer of sparsely distributed SATB + cells (layers 2, 3) located farthest from ventricular zone-like formations (VZ), with TBR1 + cells located closest to VZ. In contrast, in perfused organoids we observed homogenous distribution of CTIP2 + cells throughout the tissue, together with populations of TBR1 + and SATB2 + cells, showing no apparent layering. Previous studies^77,78^ provide evidence that, unlike in rodents, human RG cells can give rise not only to projection (excitatory) neurons but also to GABAergic inhibitory interneurons. In all three conditions, glutaminase (GLS), an enzyme expressed in glutamatergic (excitatory) neurons, and GAD67, an enzyme expressed in GABAergic (inhibitory) neurons were expressed as populations of cells with minimal spatial overlap. The extent of arborization in GLS + and GAD67 + neurons was similar to that in BLBP + and DCX + cells. In particular, we observed bundles of densely packed GAD67 + fibers in perfused organoids, while in control organoids we only detected populations of cells displaying diffuse GAD67 with no cellular processes evident (Fig. 5f, Supplementary Fig. 10c).

Taken together, our results indicate that the micro-capillary perfusion enhances proliferation of developing organoids and stimulate the proliferation of a radial glia network as well as excitatory and inhibitory microcircuits.

### Long-term perfusion of liver tissue constructs

**Fig. 6:**
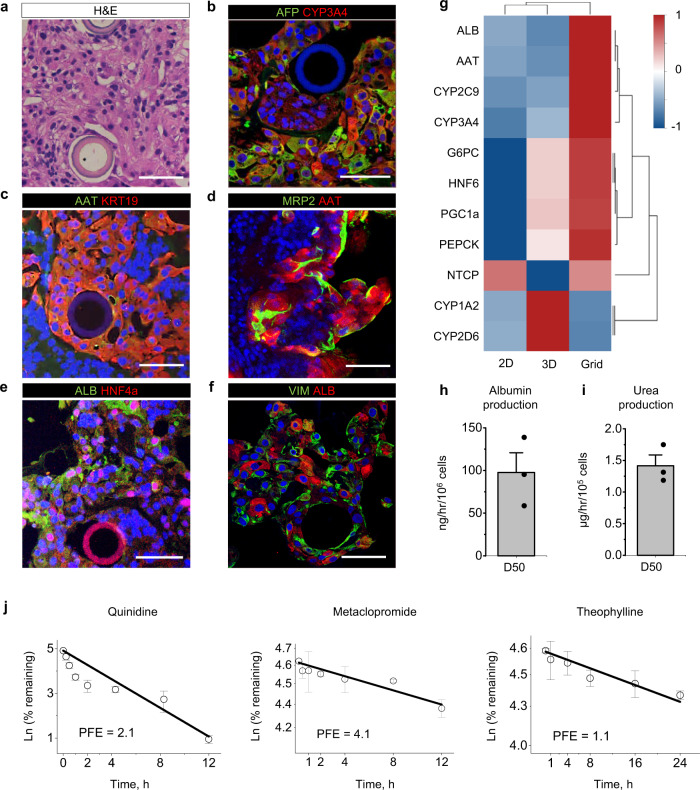
Functional improvement in perfused liver constructs. **a** Hematoxylin and eosin of a transverse section of liver tissue construct with hepaptocyte-like morphology and no visible indication of apoptosis. **b** Immunofluorescence images showing presence of Alpha Fetoprotein (AFP) and hepatic P450 cytochrome CYP3A4, **c** Alpha-1 antitrypsin (AAT) and Cytokeratin 19 (KRT19). **d** Multiple drug resistance-associated protein (MRP2) and α1-antitrypsin (AAT), **e** Basic hepatic markers albumin (ALB) and hepatic nuclear factor (HNF4α) expression (**f**) Vimentin (VIM) and albumin (ALB). Scale bar 70 µm. Representative images on (**a**) through (**f**) were acquired in three independent experiments; all experiments produced similar results. **g** Heatmap representation of fold change gene expression levels normalized to control 2D cell culture and compared to standard hepatic organoids and perfused liver constructs; rows are centered and scaled. Gene expression data for hepatic 2D culture and hepatic organoids are from Kumar et al.^103^. Color bar represents normalized fold change (dimensionless) in the expression of the corresponding genes. **h** Perfused hepatic constructs produced albumin (97.6 ± 23.1 ng/h/10^6^ cells, *n* = 3) and urea (**i**) (1.4 ± 0.2 µg/h/10^6^ cells, *n* = 3). **j** Quantification of drug metabolism in perfused hepatic constructs. Based on the data for Quinidine (left, *n* = 3) Metaclopramide (middle, *n* = 3) and Theophylline (right, *n* = 3), prediction of human in vivo drug clearance was done for the corresponding drugs based on well-stirred model, with calculated predicted fold error (PFE) shown. Data are derived from independent experiments and are represented as mean ± SEM.

To demonstrate the versatility of this platform, we went on to assess the differentiation of hPSC-derived liver progenitors in perfused grids. hPSC were differentiated for 8 days in conventional 2D culture, followed by spheroid generation, seeding into the chip and perfusion for an additional 32 days. As was the case with neural differentiation, cells merged over time into a continuous tissue (Supplementary Fig. 11a), with histological analysis identifying tightly packed cells with an eosinophilic and clear vacuolated cytoplasm reminiscent of hepatocytes, with no evidence of apoptotic bodies or necrosis throughout the tissue (Fig. 6a). The expression of many hepatocyte-specific genes was upregulated in perfused tissue constructs compared to both control hepatic organoids and 2D hepatocyte differentiation, including hepatocyte nuclear factor 6 (HNF6), Na^+^/taurocholate co-transporting polypeptide (*NTCP*), albumin (ALB), alpha1-antitrypsin (AAT) and two major cytochrome *P450* enzymes CYP2C9 and CYP3A4 (Fig. 6g). The presence of CYP3A4 as well as of the hepatocyte progenitor marker AFP were confirmed at the protein level in the perfused sample by immunohistochemistry (Fig. 6b). Interestingly, the two major gluconeogenesis enzymes phosphoenolpyruvate carboxykinase (PEPCK) and glucose 6-phosphatase alpha (G6PC) were also expressed at higher levels in the perfused constructs, compared to control organoids (1.6- and 1.3- fold higher expression, respectively) (Fig. 6f).

We next assessed whether non-parenchymal cells in the developing liver, such as cytokeratin 19 (*KRT19*)-expressing cholangiocytes, which contribute to bile secretion and hepatocyte survival^79^, were also present in our perfused culture system. These cells are generated in-vivo from hepatoblasts surrounding the portal veins, while hepatoblasts located away from portal vein areas differentiate into hepatocytes^80^. We observed a similar localization pattern of KRT19 + cells, with such cells tightly surrounding every vessel in the microfluidic grid (Fig. 6c, Supplementary Fig. 11b, c), while the hepatocytic markers HNF4α^81^ was expressed in cells scattered in the inter-vessel space (Fig. 6e). The perfused liver constructs were also metabolically active, producing albumin (Fig. 6e, f, h) and urea (Fig. 6j) at a rate of 97.6 ± 23.1 ng/h/10^6^ cells and 1.4 ± 0.2 µg/h/10^6^ cells, respectively.

### Perfused liver tissue constructs predict human in vivo clearance of slowly metabolizing drugs

Prediction of human pharmacokinetics is of key importance for safe and efficacious drug treatment. However, despite constantly evolving in vitro models of drug metabolism in humans^82,83^, including recently developed relay methods^84–86^ and 3D culture devices^87,88^, the assessment of clearance of low-turnover compounds remains a major challenge^87^, even with the use of primary human hepatocytes^89,90^, due to inability of such in vitro assays to maintain their enzymatic activity for sufficiently long time periods to estimate the clearance of slowly metabolized compounds^91^.

To address this limitation, we employed our perfused hepatic constructs to predict human in vivo clearance of quinidine, metoclopramide and theophylline, compounds which are metabolized by P450 enzymes CYP3A4, CYP2D6 and CYP1A2 respectively, and which are commonly used in liver drug clearance studies (Table 1 and Fig. 6k).

**Table 1:** Summary of drug clearance experiments

Compound	Enzyme	Turnover	Predicted clearance (ml/min/kg)	Observed clearance (ml/min/kg)	Fold prediction error (FPE) (predicted/observed)
Quinidine	CYP3A4	Intermediate	10	4.7	2.1
Metoclopramide	CYP2D6	Intermediate	1.5	6.2	0.2
Theophylline	CYP1A2	Slow	0.8 (1.5)	0.77	1.0 (1.9)

The numbers in parentheses represent results from a second application of theophylline after a complete change of incubation medium.

The clearance of one of the two compounds with intermediate rate of metabolism, quinidine, was predicted well (10 ml/min/kg, FPE = 2.1), representing only an approximately two-fold difference from its observed in vivo clearance value^92^. Theohpylline, which is known to have a low clearance rate in vivo^93^, was expected to represent the greatest challenge to our platform. Its clearance was predicted very well, estimated as 0.8 ml/min/kg in comparison to a reported value of 0.77 ml/min/kg in vivo (FPE = 1.0). Moreover, the successive application of theophylline to the constructs resulted in a similarly well predicted clearance (1.5 ml/min/kg, FPE = 1.9). This demonstrates that hPSC-derived hepatic constructs can achieve high in vitro-in vivo correlation (IVIVC) for low-clearance compounds, suggesting the use of perfused hepatic constructs as in-vitro models for the evaluation of human drug metabolism.

Taken together, our results confirm the feasibility of using this synthetic micro-vascularization approach as a generic strategy to build large and long-term functional, perfused in-vitro tissues.

## Discussion

Here, we demonstrated an integrated 3D culture platform which provides a physiologically relevant micro-perfusion for engineered tissues, resulting in enhanced tissue growth and differentiation compared to previously reported in-vitro tissue vascularization strategies.

We showed that microvascular networks could be created using 2-photon hydrogel polymerization, and that this technology can be used to achieve micro-vasculature with previously unreported accuracy, resolution and scale. A significant limitation of current photo-polymerizable hydrogel materials is the significant swelling of the material, which prevents the robust, leak-free interface between printed structures and microfluidic perfusion systems. Our development of a non-swelling photo-polymerizable material formulation was a critical component to overcome this limitation, and enabled the printing of 3D soft microfluidic systems which could be reliably perfused over multiple weeks. The exchange of nutrients and oxygen as well as the removal of waste products was achieved via simple diffusion as the printed vascular network is permeable to water-soluble molecules and gases. The direct fabrication of capillaries of a defined topology delivers an unprecedented control over tissue perfusion parameters, and the design of the vascular network is highly flexible and can be adapted to more complex geometrical and structural requirements. To provide a complete extracellular milieu with structural 3D support to the growing engineered tissue, the space around the perfused vessels was filled with hydrogel. In the experiments presented here, the hydrogel component consisted of the commonly used proteinaceous matrix Matrigel, however the platform can also readily accommodate without any additional modifications the use of other naturally derived matrices such as collagen, as well as synthetic artificial extracellular matrices such as poly(ethylene) glycol PEG^94^ or alginate^95^. This technology provides a reliable fluidic coupling between the microfluidic grid and the host perfusion device, such that continuous peristaltic pump-driven perfusion is possible. By integrating these printed microfluidic grids into a perfusion system, we were able to show that large-scale (>15mm^3^) fully perfused neural and liver tissues could be generated with this platform.

Our experiments with neural tissue demonstrate that the differentiation trajectory of cells in this perfused system is significantly enhanced. While control organoids remained largely in a pluripotent state, scRNAseq analysis revealed that perfused tissue rapidly differentiated towards the neural fate, together with a switch from glycolysis to oxidative phosphorylation. Imaging and flow cytometry confirmed that this tissue was highly viable, and immunohistochemistry showed markers of neural differentiation, which were absent in the non-perfused sample, as well as hallmarks of epithelial to mesenchymal transition. This was underscored by our observations that the lack of active micro-perfusion of the engineered tissue construct triggered a stress response in the cells within the inner core of the tissue. The accelerated differentiation of tissues upon perfusion is thought to be due not only to increased availability of nutrients and oxygen but also to the rapid diffusion of differentiation factors within the tissue via the tightly spaced capillary network.

The possibility of long-term perfusion within this platform was also demonstrated by the maintenance of viable engineered liver tissue exhibiting enhanced phenotypic and functional features compared to standard 2D and 3D organoid culture. This platform could also predict in vivo human metabolism of the slowly metabolized compound theophylline, making this culture technology ideally suited to this type of drug discovery application.

In addition to generating large functional tissue constructs, we employed our perfusion platform for growing single cortical organoids. In these perfused organoids, we confirmed the prevention of apoptosis in the inner core of the organoids, and observed enhanced neuronal and glial arborization over the course of long-term differentiation. A combination of factors, including oxygenation, the presence of extracellular matrix components as well as the dynamics of mechanical forces have been shown to play a role in organoid morphogenesis^96^. In this regard, the apparent lack of zonation in our liver tissue constructs as well as the absence of layered SATB2/TBR1/CTIP2 expression could be a result of the physical and geometric characteristics of the perfusion network. Indeed, the tightly spaced capillaries arranged into a regular structure of the grids provide a uniform supply of oxygen and nutrients, as confirmed by the absence of apoptosis or hypoxia in the perfused tissues, as well as a uniform distribution of mechanical forces at the macroscopic scale. Combined with the absence of external mechanical forces induced by shaking or mixing in conventional organoid cultures, our culture approach effectively eliminates any exogenous cues promoting symmetry breaking in the tissue development. The use of larger inter-capillary distances could potentially restore zonation in the perfused liver tissue or cortical layering in perfused cerebral organoids, and, more broadly, the introduction of spatially engineered physical and chemical cues in this platform could be used to impose defined developmental patterning. In addition, further steps towards increasingly biomimetic tissue constructs could involve the combination of our approach with the seeding of vascular cells within the channels, which could be engineered to be degradable, as well as the incorporation of vascular cells in co-culture within the perfused tissue constructs.

Overall, the 3D soft microfluidic technology presented here overcomes one of the major challenges in engineering tissues and organoids: the lack of tissue perfusion from the initiation of tissue growth, and enables the generation of large engineered tissues which are vascularized from within and their maintenance over long periods of time. In applications such as disease modeling and drug development, such a highly defined synthetic perfusion system would be beneficial in avoiding the complexity and variability introduced by exogenous angiogenesis-driven vascularization. While the current implementation of the platform does not recapitulate features of in vivo vascular networks such as adaptive vascular remodeling or selective blood brain barrier interactions, it addresses the major problem of oxygen, nutrient and growth factor and small molecule supply as well as of waste removal, allowing to generate viable tissues beyond currently available dimensions. The incorporating of endothelial vasculature with synthetic capillaries could be implemented, where the hydrogel micro-vascularization could provide a temporary tissue support during the time required for angiogenesis-driven capillarization to establish a perfusable network. We expect that this approach is widely applicable in overcoming the current size limitations of bio-printed tissues and provides a technological foundation for the development of perfusable in vitro models of increased complexity and scale.

## Methods

### Human PSC culture

Human PSCs were cultured in Matrigel (356277, Becton Dickinson) coated 6 well plates up to 60-70% confluency. Passages were performed by a 3 min treatment of Dispase II (D4693, Sigma) at 37 °C, followed by 2–3 PBS washings at RT. 1 mL of E8-Flex medium (A2858501, Thermo Scientific) was added and the colonies were scraped and gently pipetted 4–5 times through 1 ml plastic tip to break the colonies. The colony suspension was then diluted at 1:5 ratio and plated to a Matrigel-coated wells in 2 mL of E8Flex medium supplemented with 10 μM Rock inhibitor (Y-27632, Hellobio) for 24 h. The medium was then replaced by 2 mL of fresh E8-Flex medium and incubation was continued for 48 h, at which point the colonies usually reached 60–70% confluency and were ready for next passage.

### Human PSC- derived cerebral organoids and perfused cerebral tissue

We adapted the protocol by Lancaster et al.^97^ to our experimental conditions. Upon reaching a confluency of 60–70% hPSCs were dissociated by treatment the colonies with 250 µl Accutase (A1110501, Gibco) for 7 min at 37 °C and re-suspended in E8Flex medium containing 10 μM Rock inhibitor.

Organoids were generated in U-bottom 96-well plates (#351177, Falcon). The plates were rinsed with Anti-Adherence Solution (#07010, Stemcell Technologies) and cells were plated at 9000cells/well density. Plates were spun at 300rcf at RT and left in CO2 incubator for 24 h for hPSC spheroid aggregation, after which culture medium was replaced by fresh E8-Flex medium, without Rock inhibitor and changed afterwards every 2 days. At day 2, the spheroids were embedded in growth factor reduced (GFR) Matrigel (354230, Becton Dickinson) and kept in 6-well plates, pre-rinsed with Anti-Adherence Solution in CO2 incubator. At day 6, neuronal induction was started by replacing E8-Flex medium with DMEM/F12 medium (31330038, Gibco) containing 1% MEM-NEAA (11140035, Gibco), 1% Glutamax (35050038, Gibco), 1% Pen-Strep (15140122, Gibco), 0.5% N2 supplement (17502048, Gibco) and 1ug/ml Heparin (H3149, Sigma) (neural induction medium). Day 8 spheroids were used for characterization.

For the formation of perfused tissue, at day 0, we first generated micro-hPSC spheroids using 24-well Aggrewell plates (34411, Stemcell Technologies) following a protocol supplied by the manufacturer. Specifically, we seeded Aggrewells with hPSCs to obtain 350-400 cells per micro-well. After 24 h, spheroids formed and the medium was replaced by fresh E8-Flex without Rock inhibitor. At day 2, the spheroids typically reached a diameter of 180-200um, and were harvested and re-suspended in ice cold GFR Matrigel at a density of 3500 spheroids per 200-250ul GFR Matrigel. The grids were placed under stereomicroscope in a 35 mm petri dish on ice and seeded with the spheroid suspension at final density of ~1800 spheroids per grid. 200 µl plastic tips pre-chilled on ice were used to dispense the suspension under stereomicroscope. Seeding was performed in several stages in order to allow dispensed spheroids to settle into the grids. After seeding, the grids were kept in a CO2 incubator for 40–50 min to allow Matrigel polymerization, after which the grids were placed in a perfusion chip and perfusion was started. E8-Flex/PenStrep medium was changed every 2 days (3–4 ml per grid). At day 6, E8-Flex medium was replaced by neural induction medium for 2 days. At day 8, the grids were extracted, and the tissue used for characterizations.

### Immunofluorescence analysis of cerebral tissue samples

Samples were fixed with 4% paraformaldehyde (158127, Sigma-Aldrich) for 24–36 h at 4 °C and washed by 3 incubations in PBS for 15–20 min at room temperature. Fixed tissue was sectioned either by embedding in low melting point agarose and sectioning on vibratome (Leica VT1000S) into 100–150 µm sections or cryopreserved in OCT (6502, Thermo APD Consumables) overnight at 4 °C, re-embedded into fresh OCT, frozen in isopropanol/dry ice slurry, cut into 50 µm sections on cryotome (Leica CM1850) and affixed on SuperFrost Plus (10019419, Thermo Scientific) microscope glass slides.

Sections were incubated in a permeabilization and blocking solution of 0.3% Triton X (A4975, PanREAC AppliChem) and 3% BSA (A7906, Sigma) in PBS for 24 h at 4 °C. Primary antibodies were diluted in the permeabilization and blocking solution and applied to sections for 24 h at 4 °C, after which three PBS washes were performed over another 24 h period. Secondary antibodies and Hoechst were also diluted in the permeabilization and blocking solution and applied to sections overnight at 4 °C, followed by washing in PBS 3-4 times over another 24 h. Detailed antibody information is given in Supplementary Table 1. Stained agarose-embedded sections were stored in 2 mM sodium azide solution in PBS. Cryosections were mounted in Fluoromount-G medium and stored at 4 °C.

### Human PSC-derived perfused hepatic tissue

All liver differentiation experiments were performed with the H3CX hPSC line previously generated^98^. H3CX is a hPSC line (Sigma 0028, Sigma-Aldrich) genetically engineered to overexpress 3 transcription factors HNF1A, FOXA3 and PROX1 upon Doxycycline induction, which allows for rapid generation of hepatocyte-like progeny. H3CX cells were expanded feeder-free on Matrigel (BD Biosciences)-coated plates in E8 or E8 Flex (Thermo Fisher Scientific). HC3X cells were differentiated towards HLCs as previously described^99^. Briefly, HC3X cells were dissociated to single cells using StemPro™Accutase™ Cell dissociation Reagent (Thermo Fisher Scientific) and plated on Matrigel-coated plates at ±8.75 × 10^4^ cells/cm^2^ in mTeSR medium (Stem Cell Technologies) supplemented with RevitaCell (Thermo Fisher Scientific). When cells reached 70–80% confluence, differentiation was performed during 40 days in liver differentiation medium (LDM) containing to comprise 500 ml total volume: 285 ml of DMEM low glucose (Invitrogen 31885023), 200 ml of MCDB-201 solution in water (Sigma M-6770) adjusted to pH 7.2, 0.25× of Linoleic acid—Bovine serum albumin (LA-BSA, Sigma L-9530), 0.25× of Insulin–transferrin–selenium (ITS, Sigma I-3146), 50 U of Penicillin/Streptomycin (Invitrogen 15140122), 100 nM of l-ascorbic acid (Sigma A8960), 1 μM dexamethasone (Sigma D2915) and 50 μM of β-mercaptoethanol (Invitrogen 31350010). Differentiation medium was supplemented with 0.6% dimethylsulfoxide (DMSO) during the first 12 days of the culture. 2.0% DMSO and 3x concentrate of non-essential amino-acids (NEAA) was added to LDM medium between days 12–13, and from day 14 until the end of differentiation 20 g/L glycine was added to LDM medium supplemented with NEAA. Differentiation was performed in presence of the following factors: day 0–1: 100 ng/ml Activin-A and 50 ng/ml Wnt3a, day 2–3: 100 ng/ml Activin-A, day 4–7: 50 ng/ml BMP4, day 8–11: 20 ng/ml FGF1, and 20 ng/ml HGF during the rest of differentiation. Doxycycline (5ug/ml) was applied from day 4 until the end of differentiation. All cytokines were purchased from Peprotech.

### RNA extraction and quantitative reverse-transcription PCR

RNA extraction was performed using TRIzol reagent (Invitrogen) following manufacturer’s instructions. At least 1 µg of RNA was transcribed to cDNA using the Superscript III First-Strand synthesis (Invitrogen). Gene expression analysis was performed using the Platinum SYBR green qPCR supermix-UDG kit (Invitrogen) in a ViiA 7 Real-Time PCR instrument (Thermo Fisher Scientific). The sequences of all used RT-qPCR primers are listed in Supplementary Table 2. The ribosomal protein L19 transcript (RPL19) was used as a housekeeping gene for normalization.

### Histology

Samples were fixed with 4% (w/v) paraformaldehyde (PFA, Sigma-Aldrich) overnight at 4 °C, washed three times with PBS and submerged in PBS-sodium azide (0.01% v/v) solution at 4 °C until embedded in paraffin. Hydrogel sections (5 µm) were prepared using a microtome (Microm HM 360, Marshall Scientific.) For Hematoxylin and Eosin (H&E) staining, sections were treated with xylene solution to remove the paraffin, and gradually rehydrated in ethanol (100–70%, v/v). H&E staining was performed by submerging rehydrated hydrogel sections in Harris Hematoxylin solution, acid alcohol, bluing reagent and Eosin-Y solution by order. Stained samples were dehydrated with ascending alcohol series, washed in xylene solution, and mounted with DPX mountant (Sigma-Aldrich).

### Immunofluorescence analysis of liver tissue samples

Following deparaffinization in xylene and rehydration in descending alcohol series, heat-mediated antigen retrieval was performed by incubating hydrogel sections in Dako antigen retrieval solution (Dako) for 20 min at 98 °C. This step was followed by cell permeabilization with 0.01% (v/v) Triton-X (Sigma-Aldrich) solution in PBS, for 20 min. Samples were then incubated with 5% (v/v) Goat or Donkey Serum (Dako) for 30 min. Primary antibodies diluted in Dako antibody diluent solution, were incubated overnight at 4 °C, followed by washing steps and incubation with Alexa-coupled secondary antibody (1:500) and Hoechst 33412 (1:500) solution for 1 h at room temperature. Finally, samples were washed in PBS, and mounted with Vectashield antifade mounting medium (Vector Laboratories). Stained sections were imaged using laser scanning confocal microscope (LSM 880, Zeiss, Germany), and image processing were performed on ZEN Blue software (Zeiss, Germany).

### Functional assessment of perfused hepatic tissue at day 50

Albumin secretion rate was quantified using the human albumin ELISA quantitation kit (Bethyl Laboratory) according to manufacturer guideline. Urea secretion rate was analyzed in freshly prepared medium, after 12 h and 24 h of culture using the QuantiChromTM Urea Assay Kit (BioAssay Systems DIUR-500) according to the manufacturer’s instructions.

To assess CYP450 biotransformation capacity, perfused hepatic constructs were treated with quinidine (1 µM), metoclopramide (1 µM) and theophylline (1 µM) in order to test the activity of CYP3A4, CYP2D6 and CYP1A2 respectively. LC-MS/MS analysis was used to determine the rate of drug degradation by hepatic constructs and predict human in vivo clearance of the drugs.

Drugs were prepared as 1 M stock solutions in pure water (18.2MOhm, filter sterilized) and further diluted in culture medium. Only one drug was applied at a time. Medium samples (40 µL) were collected at time points best representing expected clearance of the corresponding drug (Fig. 6k), quenched by addition of acetonitrile (40 µL) containing 50 nM carbamazepine used as a reference drug, and stored at −80 °C for further analysis. Prior to analysis, samples were further diluted 2-3x with HPLC-grade water.

Samples were injected onto a Kinetics C18, 5 μm, 30 × 2.1 mm analytical column (Phenomenex, Torrance, CA, USA) at ambient temperature. The LC-MS/MS system consisted of a Transcend 1250 pump and a LX-2 Thermo Cohesive duplexing apparatus (Thermo Scientific, Waltham, MA, USA) linked to a CTC PAL auto-injector (Leap Technologies, Carrboro, NC, USA) and QTRAP 5500 mass spectrometer (AB Sciex, Foster City, CA, USA). Drug concentrations in culture media samples were determined using reverse-phase liquid chromatography with a solvent system consisting of 0.1% formic acid in water (mobile phase A) and acetonitrile with 0.1% formic acid (mobile phase B). A gradient of mobile phase B remained constant at 5% over 10.2 s, then increased from 5% to 95% over 4.8 s and remained at 95% mobile phase B for 120 s, then decreased back to 5% mobile phase B over 15 s with equilibration for 20.4 s prior to the next sample injection (5 μL sample injection volume). The mobile phase flow rate used was 0.9 mL/min. Detection of drugs (retention time ~1.5 min) and the internal standard carbamazepine was achieved in the positive ion mode by multiple reaction monitoring for the transition [M + H] + m/z 325.2–307.1, 299.8–226.9 and 181.1–124.1 (quinidine, metoclopramide and theophylline respectively) and [M + H] + m/z 237.0–194.0 (carbamazepine). Samples were quantified against a calibration curve spanning a 2000-fold concentration range (1–2000 ng/mL). Peak areas for the drugs and internal standard were obtained using Analyst 1.6 (AB Sciex, Foster City, CA, USA), and the peak area ratio (drug/internal standard) was used for quantitation.

Drug clearance was predicted based on well-stirred model^92,100^. Briefly, a drug half-decay time was calculated from the linear regression slope (*k*) of natural logarithm of normalized concentration of drug remaining (% drug remaining) in medium samples versus incubation time. For all compounds the accuracy of linear fit (adjusted *R*^2^) was >0.97. CL_lint, app_, an apparent hepatocyte in vitro intrinsic clearance was estimated as:

1 $${{{{{{\rm{CL}}}}}}}_{{{{{{\rm{int}}}}}},{{{{{\rm{app}}}}}}}=\frac{{{{{{\rm{ln}}}}}}(2)}{{t}_{1/2}}\times \frac{{{{{{\rm{V}}}}}}}{{{{{{\rm{N}}}}}}},{mL}/\min /{million}\,{cells}$$

where V is a volume of the incubation, N is number of cells.

Then, CL_int, app_ was scaled to in vivo units:

2 $${{{{{{\rm{CL}}}}}}}_{{{{{{\rm{int}}}}}},{{{{{\rm{scaled}}}}}}}={{{{{{\rm{CL}}}}}}}_{{{{{{\rm{int}}}}}},{{{{{\rm{app}}}}}}}\times {{{{{\rm{SF}}}}}}\times {{{{{\rm{LW}}}}}},{{{{{\rm{mL}}}}}}/{{{{{\rm{min }}}}}}/{{{{{\rm{kg}}}}}}$$

where SF = 107 million cells/g liver, LW = 26 g liver/kg body weight.

Predicted in vivo hepatic clearance (CL_H_) was calculated as

3 $${{{{{{\rm{CL}}}}}}}_{{{{{{\rm{H}}}}}}}=\frac{{{{{{{\rm{Q}}}}}}}_{{{{{{\rm{H}}}}}}}\times {{{{{{\rm{CL}}}}}}}_{{{{{{\rm{int}}}}}},{{{{{\rm{scaled}}}}}}}}{{{{{{{\rm{Q}}}}}}}_{{{{{{\rm{H}}}}}}}+{{{{{{\rm{CL}}}}}}}_{{{{{{\rm{int}}}}}},{{{{{\rm{scaled}}}}}}}},{ml}/\min /{kg}$$

where human blood liver flow (Q_H_) estimated as 21 ml/min/kg.

### Image acquisition and analysis of cerebral tissue samples

Fluorescence images were obtained using a confocal microscope (Leica SP8 DIVE, Leica Microsystems) equipped with 10x NA0.4 dry objective. Acquisition parameters were kept constant for all samples obtained in the same experiment. Image processing was performed using Fiji/ImageJ (NIH) and custom-written ImageJ Java plugin. At the data preprocessing stage, image stacks were collapsed by maximum intensity projection. Resulting images were translated to 8-bit lookup table representation, manually cleaned from grid structure elements and thresholded by replacing all sub-threshold pixels with black (zero value) pixels. Lookup tables of the images were then remapped from [Threshold..255] back to full [0..255] range. Threshold values were chosen manually for Hoechst and marker channels for each image in order to remove background from the images. For all markers except HIF1α, total protein expression levels in a tissue were quantified as a relative number of cells expressing the protein:

4 $$\ {Protein}\,{expression}=\frac{{{Marker}}^{+}{cell}\,{number}}{{Total}\,{cell}\,{nnumber}}x100\%$$

On the assumption that cell size is constant across the samples, the relative number of cells was estimated as a ratio of areas occupied by cells on marker and Hoechst channels. Corresponding areas were calculated by counting all non-zero pixels on marker and Hoechst channels. For statistical comparison, results for each marker were pooled from all experiments.

As the HIF1α marker is constitutively expressed in cells under normoxic conditions, its expression level in a tissue was estimated as the average fluorescence intensity on the marker channel. Threshold values were kept constant for organoids, non-perfused and perfused samples obtained in the same experiment; expression values obtained in the experiment were normalized to organoid value and normalized data from different experiments were averaged.

### Statistical analysis

All results are expressed as mean ± SEM. Statistical comparisons were analyzed using unpaired two-tailed Student’s *t*-test with a 95% confidence interval (Origin Pro v9.5, OriginLab Inc.). Significance was marked on plots by *, ** and *** for *p* < 0.05, *p* < 0.01, and *p* <  0.001 correspondingly. If not stated otherwise, the statistical significance was assessed for comparisons of perfused and non-perfused samples to organoid control samples and denoted by “p_Control_”. No statistical method was used to predetermine sample size. Sample sizes for differentiation experiments and drug clearance studies were based on sample sizes generally used in the similar studies. All collected data were used for the analysis. In the long term neural differentiation experiments, organoids were randomly allocated between the three experimental experimental conditions. The study was not blinded.

### Organoid dissociation and single-cell RNA sequencing

For dissociation, the microfluidic grids of non-perfused and perfused samples were removed from the perfusion chips and, along with the control organoids, placed temporarily in DMEM/F12 media in independent 15 mL Falcon tubes until all three samples were ready for the next step (time <5 min). The DMEM was then replaced with 1 mL of pre-warmed TrypLE Express (12605010, Gibco) at 37 °C. The samples were transferred to a warm water bath at 37 °C for 7.5 min with gentle agitation every 1 min after the first 3 min. A visual inspection was performed using an inverted microscope to ensure complete dissociation of tissues and the presence of single cells. The 1 mL solution was introduced to 9 mL of DMEM supplemented with 20% FBS for TrypLE Express neutralization, and centrifuged at 500 g for 5 min. The pellet was re-suspended in 200 uL of N2B27 media and put on ice. This dissociation protocol yielded an average viability of above 80% across all samples.

Library preparations for the scRNAseq was performed using 10X Genomics Chromium Single Cell 3' Kit, v3 (10X Genomics, Pleasanton, CA, USA). The cell count and the viability of the samples were accessed using LUNA dual fluorescence cell counter (Logos Biosystems) and a targeted cell recovery of 6000 cells was aimed for all the samples. Post cell count and QC, the samples were immediately loaded onto the Chromium Controller. Single-cell RNAseq libraries were prepared using manufacturers recommendations (Single cell 3' reagent kits v3 user guide; CG00052 Rev B), and at the different check points, the library quality was accessed using Qubit (Thermo Fisher) and Bioanalyzer (Agilent). Single cell libraries were sequenced using paired-end sequencing workflow and with recommended 10X; v3 read parameters (28-8-0-91 cycles). The data generated from sequencing was de-multiplexed and mapped against human genome reference using CellRanger v3.0.2.

### Single Cell RNA sequencing data processing

We have sequenced 4818, 5453, and 2804 cells for non-perfused, perfused and control organoids samples respectively for a total of 13,075 cells, which were reduced after QC steps to 8625 cells with an average of 1852 detected genes per cell. Data processing and subsequent steps were performed using the Seurat^101^ tool for single-cell genomics version 3 in R version 4.0.3. A filtering step was performed to ensure the quality of the data, where the counts of mitochondrial reads and total genes reads were assessed. Cells with >15% of identifiable genes rising from the mitochondrial genome were filtered out. Similarly, cells having fewer identifiable genes than 200 (low quality) and above 7500 (probable doublets) were filtered out. Data normalization was performed, followed by the identification of 2000 highly variable genes using the FindVariableFeatures(). S-phase and G2M-phase cell cycle regression was performed to allow cell clustering purely on cell identity and fate, which otherwise was biased by cell cycle phases. Auto scaling of the data was performed and described using principal component analysis (PCA) using the RunPCA() function.

### Correlation analysis

Correlation heatmap between samples was performed in R using the top 100 marker genes for each sample using the FindAllMarkers() function followed by the selection of the top 100 marker genes using the top_n() function with *n* = 100 and wt = avg.logFC. The correlation analysis was generated using the cor() function using the Pearson method and the heatmap using the heatmap.2() function in R.

### Data clustering

Graph-based clustering using the FindNeighbors() function (using top 15 principal components (PCs)) and FindCluster() function (resolution = 0.5) was performed to group cells based on their transcriptional profiles. No batch correction was performed on the dataset. Data visualization was performed using the Uniform Manifold Approximation and Projection (UMAP) dimensionality reduction technique using the RunUMAP package while employing the top 15 PCs identified in the previous PCA step. Cluster annotation was based on hallmark genes to identify Neuroepithelial cluster (PAX6, PAX7, PAX3), Pluripotent-Neuroepithelial transitioning (P-NE) cells (WNT4, IRX1, IRX2), Proliferating cells (CDC6, PTN, MKI67, TOP2A), medium mitochondrial gene content and low glycolysis pluripotent cells (MKI67, TOP2A, with the expression of NANOG, POU5F1, DPPA4), Pluripotent cells (NANOG, POU5F1, DPPA4), highly glycolytic pluripotent cells that link to the Cycling pluripotent and Pluripotent cells but include the expressions (LDHA, ENO1, HK2), a cluster undergoing EMT/migration with hypoxic markers (EPCAM, VIM, TWIST2, VEGFA) and finally a stressed cluster (FOS, HIF1α, JUN). One cluster was manually removed due to very low unique molecular identifier (UMI) count, after which the data was reprocessed to account for the removal of the cluster.

### Pseudotime trajectories

Trajectory analysis was performed using Monocle 3^102^ on R by employing the monocole3 and SeuratWrappers libraries. The Seurat obtained object containing all combined samples was passed to the Monocle 3 pipeline using the as.cell_data_set() function followed by processing using the cluster_cells() function. The pseudotime trajectory was inferred by first using the subset function on the only partition detected, followed by the learn_graph() function. Cells were colored in their inferred pseudotime using the which.max() function with the FetchData() (AVP) function, followed by the order_cells() function with the root_cell = max.avp. The UMAP superimposed with inferred pseudotime trajectory was generated using the plot_cells() function.

### Gene-set analysis and hierarchical clustering

Gene-sets were created using the AddModuleScore() function in R. three gene-set were created to explore the dataset, pluripotency gene-set (*POU5F1, NANOG, CDH1*), glycolysis gene-set (*ALDOA, BPGM, ENO1, ENO2, GPI, HK1, HK2, PFKL, PFKM, PGAM1, PGK1, PKM, TP11*), and a neural progenitor gene-set (*PAX3, PAX6, PAX7, OTX2, CDH2*). The % mitochondrial genes were obtain as evaluated by the Seurat pipeline. Each cell was evaluated and scored and its expression plotted on UMAPS. Cluster level hierarchical clustering of the gene-set scores and % mitochondrial genes was employed using R package heatmap.2 after scaling the expression across rows (clusters).

### Design and fabrication of soft microfluidic grids

The microfluidic grids were designed as a multitude of parallel capillaries stemming from a common reservoir. The dimensions of the grid (2.6 × 2.6 × 1.5 mm) were chosen as a compromise between size and the fabrication time. The grids were micro-fabricated on custom baseplates using high-resolution 3D printer (Photonic Professional GT2, Nanoscribe GmbH) equipped with 2-photon femtosecond laser. CAD model of the capillary grid was pre-processed by DeScribe software (Nanoscribe GmbH) to produce a printing job with defined printing parameters. A custom-formulation photopolymerizable resin was used to print microfluidic grids which contained 2% 2-Benzyl-2-(dimethylamino)-4'-morpholinobutyrophenone (Irgacure 369), 7% propylene glycol methyl ether acetate (PGMEA), 36% poly(ethylene glycol) diacrylate, MW700 (PEGDA 700), 25% pentaerythritol triacrylate (PETA), 20% Triton-X 100 and 10% water. Irgacure 369 was purchased from Tokyo Chemical Industry Ltd, the other components were from Sigma-Aldrich. Given the chosen printing parameters and composition of the resin, it took 3 h to produce one grid. However, the printing time can be drastically reduced by increasing photo-sensitivity of the resin, using 2-photon systems equipped with more powerful laser and/or by optimization of the microfluidic grid design.

The microfluidic grids were fabricated on custom plates 3D printed on Formlabs Form 2 printer from a biocompatible resin (Dental SG, Formlabs Inc), post-cured by UV light (Formcure station, Formlabs Inc) for 2 h at 80 °C and washed in 2-propanol for 3–4 days with daily change of the solvent. The plates had 1 mm thickness and 10 mm diameter, with 0.5 mm perfusion holes. The grid printing process was set up such that the inlets in the microfluidic grid were aligned with the perfusion holes in a baseplate. After fabrication, baseplate-grid assemblies were washed during 2 days in PGMEA, 12 days in 70% ethanol and then kept in PBS until use. The solvents were refreshed every 1–2 days and PBS was refreshed daily for the first week and every 2–3 days afterwards.

### Rheology of non-swelling photopolymer

Polymer disks (8 mm dimeter x 1 mm thickness) were prepared by casting pre-polymer between two glass plates and curing under UV light for 1 min (Formcure station, Formlabs Inc). The polymerized disks were washed 2 days in PGMEA and 3 days in 70% ethanol, and kept in water until tested. The measurements were performed on an ARES-G2 stain-controlled rheometer (TA Instruments, USA) equipped with Peltier-heated temperature controlled bottom plate. An 8 mm diameter stainless-steel plate was used. The gap was set based on the normal force. A normal load (compression) of 25 g was applied during the measurements, resulting in a measurement gap around 1 mm. The dynamic moduli were obtained by performing oscillatory shear sweeps, with frequency range resulting in stable measurements from 100 to 10 rad/s (5 points/decade) at a strain of 0.0025%, which remained in the linear regime.

For water retention tests, polymer samples (20 mm x 40 mm x 1 mm) were prepared by casting pre-polymer between two glass plates and curing under UV light for 1 min. The polymerized sheets were washed 2 days in PGMEA and 3 days in 70% ethanol, wicked with filter paper and immediately weighed. The second weighing was done after overnight baking at 75 °C. Water content was estimated as a ratio of post-baked and pre-baked mass of the sheets.

### Fabrication of perfusion chips and setting up perfusion

The perfusion chips were implemented as an assembly as shown in Supplementary Fig. 1. Two microfluidic grids were sandwiched between two polydimethylsiloxane (PDMS) custom-profiled blocks. Two metal plates clamped with screws provided a tight junction between PDMS blocks and cover slips, thereby forming fluidic channels within the chip. The chips were designed to only allow contact of the medium with PDMS blocks and cover slips, thereby isolating the flow from contact with other materials of the chip. Guiding frames were used to simplify the alignment of the PDMS blocks during the chip assembly process.

The PDMS blocks were made by casting liquid PDMS compound into custom-designed plastic molds and curing overnight at 80 °C. The plastic molds were fabricated by stereo-lithography (SLA) 3D printer Form 2 (Formlabs Inc.) using a bio-compatible Formlabs Dental SG resin. The printed molds were post-processed by thorough washing in 2-propanol and post-cured for 90 min at 80 °C (Formcure, Formlabs Inc.). Metal plates were made from 1 mm stainless steel sheet by laser cutting and stainless-steel screws were used for clamping the assembly. The guiding frames were 3D printed and cover slips were glued to the metal plates with a bio-compatible UV/heat curable epoxy (NOA 86H, Norland) to provide a final two-part setup shown in Supplementary Fig. 1d.

Two chambers of the chip, containing perfused and non-perfused grids, a peristaltic pump and a medium reservoir (50 ml Falcon tube) were connected by flexible PVC tubing (ID/OD 0.8/1.6 mm) in series (Supplementary Fig. 1e). The medium was taken from the reservoir by the pump and fed through chambers containing the non-perfused and then the perfused grid, after which the medium was sent back to the reservoir for CO_2_/O_2_ equilibration. A 0.22 um filter was connected between the two chambers of the chip to protect capillaries of the perfused grid from potential clogging with tissue debris. The medium reservoir and the chip were kept in a CO2 incubator, while the peristaltic pump remained outside to protect electronics from humidity and to ensure proper cooling. The medium reservoir and the chip were installed on a custom-made 3D printed holder (Supplementary Fig. 1f) to simplify transitions between a CO2 incubator and a laminar hood. At day 0 of an experiment, all parts of the perfusion were sterilized by ethanol and UV light in a laminar hood, connected together and filled with equilibrated medium. The microfluidic grids, seeded with hPSC spheroids, were installed in the chip (Supplementary Fig. 1d) and the chip was clamped by screws (Supplementary Fig. 1f-i). Air bubbles were removed from the system and the perfusion started at ~400 µL/min flow rate (Supplementary Fig. 1f-iii).

Multi-grid perfusion chips were designed to provide an equal multiplexed perfusion for all microfluidic grids. The fluidic inlet of the chip, via three steps of bifurcations, was split into eight fluidic channels feeding eight wells. All fluidic channels had equal length and cross-section and, therefore, equal hydraulic resistance. The multi-grid chips were fabricated by stereo-lithography (SLA) 3D printer Form 2 (Formlabs Inc.) using Formlabs Dental SG resin. The printed chips were post-processed by thorough washing in 2-propanol and post-cured for 90 min at 60 °C. 7–8 mm pieces of syringe needles (0.9 mm) were cut by hand drill/cutter (Precision drill, Proxxon Inc.) and glued into chip inlets by a biocompatible UV/heat curable epoxy glue to serve as connectors for flexible tubing. This assembly was again post-cured in FormCure station for 120 min at 80 °C to achieve complete hardening of the glue. To integrate microfluidic grids, custom profile gaskets, cut from 0.5 mm PDMS sheet by a laser cutter, were placed under the grid substrates to provide a liquid-tight contact and the substrates were fixed in place by 3D printed fasteners.

### Reporting summary

Further information on research design is available in the Nature Portfolio Reporting Summary linked to this article.

## Supplementary information

Supplementary Info

Reporting Summary

## Data Availability

The raw sequencing data, and the combined processed and metadata files generated in this study are available at GEO, under accession code “GSE181290”. All other relevant data supporting the key findings of this study are available within the article and its Supplementary Information files or from the corresponding author upon reasonable request. Source data are provided with this paper.

## Source data

Source Data

## References

[1] Kaushik G, Ponnusamy MP, Batra SK (2018). Concise review: current status of three-dimensional organoids as preclinical models: 3D organoid culture as a tool for research. Stem Cells.

[2] Ollé-Vila A, Duran-Nebreda S, Conde-Pueyo N, Montañez R, Solé R (2016). A morphospace for synthetic organs and organoids: the possible and the actual. Integr. Biol..

[3] Van Norman GA (2019). Limitations of animal studies for predicting toxicity in clinical trials. JACC: Basic Transl. Sci..

[4] Gilbert, S. F. *Developmental Biology* (Sinauer Associates, 2000).

[5] Grebenyuk S, Ranga A (2019). Engineering organoid vascularization. Front. Bioeng. Biotechnol..

[6] Nashimoto Y (2017). Integrating perfusable vascular networks with a three-dimensional tissue in a microfluidic device. Integr. Biol..

[7] Salmon, I. et al. *Engineering Neurovascular Organoids with 3D Printed Microfluidic Chips*. 10.1101/2021.01.09.425975 (2021). 10.1039/d1lc00535a 35333271

[8] Rajasekar S (2020). IFlowPlate—A customized 384‐well plate for the culture of perfusable vascularized colon organoids. Adv. Mater..

[9] Sugihara K (2020). A new perfusion culture method with a self-organized capillary network. PLoS One.

[10] Takebe T (2013). Vascularized and functional human liver from an iPSC-derived organ bud transplant. Nature.

[11] Mansour AA (2018). An in vivo model of functional and vascularized human brain organoids. Nat. Biotechnol..

[12] Cakir B (2019). Engineering of human brain organoids with a functional vascular-like system. Nat. Methods.

[13] Homan KA (2019). Flow-enhanced vascularization and maturation of kidney organoids in vitro. Nat. Methods.

[14] Zhu W (2016). 3D printing of functional biomaterials for tissue engineering. Curr. Opin. Biotechnol..

[15] Xie M (2019). Electro-assisted bioprinting of low-concentration GelMA microdroplets. Small.

[16] Cui X, Boland T, D.D’Lima D, K. Lotz M (2012). Thermal inkjet printing in tissue engineering and regenerative medicine. Recent Pat. Drug Deliv. Formulation.

[17] Dababneh AB, Ozbolat IT (2014). Bioprinting technology: a current state-of-the-art review. J. Manuf. Sci. Eng..

[18] Gudapati H, Dey M, Ozbolat I (2016). A comprehensive review on droplet-based bioprinting: Past, present and future. Biomaterials.

[19] Christensen K (2015). Freeform inkjet printing of cellular structures with bifurcations: approach freeform fabrication of bifurcated cellular structures by using a liquid support-based inkjet printing approach. Biotechnol. Bioeng..

[20] Nakamura M (2008). Ink jet three-dimensional digital fabrication for biological tissue manufacturing: analysis of alginate microgel beads produced by ink jet droplets for three dimensional tissue fabrication. J. Imaging Sci. Technol..

[21] Zhu W (2017). Direct 3D bioprinting of prevascularized tissue constructs with complex microarchitecture. Biomaterials.

[22] Ma X (2016). Deterministically patterned biomimetic human iPSC-derived hepatic model via rapid 3D bioprinting. Proc. Natl Acad. Sci. USA.

[23] Huang TQ, Qu X, Liu J, Chen S (2014). 3D printing of biomimetic microstructures for cancer cell migration. Biomed. Microdevices.

[24] Singh NK (2020). Three-dimensional cell-printing of advanced renal tubular tissue analogue. Biomaterials.

[25] Gao Q (2019). 3D printing of complex GelMA-based scaffolds with nanoclay. Biofabrication.

[26] Jia W (2016). Direct 3D bioprinting of perfusable vascular constructs using a blend bioink. Biomaterials.

[27] Xu C, Chai W, Huang Y, Markwald RR (2012). Scaffold-free inkjet printing of three-dimensional zigzag cellular tubes. Biotechnol. Bioeng..

[28] Kinoshita K, Iwase M, Yamada M, Yajima Y, Seki M (2016). Fabrication of multilayered vascular tissues using microfluidic agarose hydrogel platforms. Biotechnol. J..

[29] Roudsari LC, Jeffs SE, Witt AS, Gill BJ, West JL (2016). A 3D poly(ethylene glycol)-based tumor angiogenesis model to study the influence of vascular cells on lung tumor cell behavior. Sci. Rep..

[30] Zhang YS (2016). Bioprinting 3D microfibrous scaffolds for engineering endothelialized myocardium and heart-on-a-chip. Biomaterials.

[31] Meyer W (2012). Soft polymers for building up small and smallest blood supplying systems by stereolithography. J. Funct. Biomater..

[32] Huber B (2016). Blood-vessel mimicking structures by stereolithographic fabrication of small porous tubes using cytocompatible polyacrylate elastomers, biofunctionalization and endothelialization. J. Funct. Biomater..

[33] Kang H-W (2016). A 3D bioprinting system to produce human-scale tissue constructs with structural integrity. Nat. Biotechnol..

[34] Compaan AM, Song K, Chai W, Huang Y (2020). Cross-linkable microgel composite matrix bath for embedded bioprinting of perfusable tissue constructs and sculpting of solid objects. ACS Appl. Mater. Interfaces.

[35] Miller JS (2012). Rapid casting of patterned vascular networks for perfusable engineered three-dimensional tissues. Nat. Mater..

[36] Kolesky DB, Homan KA, Skylar-Scott MA, Lewis JA (2016). Three-dimensional bioprinting of thick vascularized tissues. Proc. Natl Acad. Sci. USA.

[37] Wu W, DeConinck A, Lewis JA (2011). Omnidirectional printing of 3D microvascular networks. Adv. Mater..

[38] Bertassoni LE (2014). Hydrogel bioprinted microchannel networks for vascularization of tissue engineering constructs. Lab Chip.

[39] Subbiah R (2021). Prevascularized hydrogels with mature vascular networks promote the regeneration of critical-size calvarial bone defects in vivo A short running title: Prevascularized hydrogels repair bone defects. J. Tissue Eng. Regen. Med..

[40] Skylar-Scott MA (2019). Biomanufacturing of organ-specific tissues with high cellular density and embedded vascular channels. Sci. Adv..

[41] Silvestri VL (2020). A tissue-engineered 3D microvessel model reveals the dynamics of mosaic vessel formation in breast cancer. Cancer Res..

[42] Smith LJ, Li P, Holland MR, Ekser B (2018). FABRICA: A bioreactor platform for printing, perfusing, observing, & stimulating 3D tissues. Sci. Rep..

[43] Sego TJ (2020). Computational fluid dynamic analysis of bioprinted self‐supporting perfused tissue models. Biotechnol. Bioeng..

[44] Murata D, Arai K, Nakayama K (2020). Scaffold‐free bio‐3D printing using spheroids as “Bio‐Inks” for tissue (Re‐)construction and drug response tests. Adv. Healthc. Mater..

[45] Applegate MB (2015). Laser-based three-dimensional multiscale micropatterning of biocompatible hydrogels for customized tissue engineering scaffolds. Proc. Natl Acad. Sci. USA.

[46] Oujja M (2009). Three dimensional microstructuring of biopolymers by femtosecond laser irradiation. Appl. Phys. Lett..

[47] Sarig-Nadir O, Livnat N, Zajdman R, Shoham S, Seliktar D (2009). Laser photoablation of guidance microchannels into hydrogels directs cell growth in three dimensions. Biophysical J..

[48] Brandenberg N, Lutolf MP (2016). In situ patterning of microfluidic networks in 3D cell-laden hydrogels. Adv. Mater..

[49] Skylar-Scott, M. A., Liu, M.-C., Wu, Y. & Yanik, M. F. Multi-photon microfabrication of three-dimensional capillary-scale vascular networks. in *Proc. SPIE 10115, Advanced Fabrication Technologies for Micro/Nano Optics and Photonics X* (eds. von Freymann, G., Schoenfeld, W. V. & Rumpf, R. C.) 101150L (SPIE, 2017).

[50] Kloxin AM, Kasko AM, Salinas CN, Anseth KS (2009). Photodegradable hydrogels for dynamic tuning of physical and chemical properties. Science.

[51] Kloxin AM, Tibbitt MW, Kasko AM, Fairbairn JA, Anseth KS (2010). Tunable hydrogels for external manipulation of cellular microenvironments through controlled photodegradation. Adv. Mater..

[52] Tibbitt MW, Kloxin AM, Dyamenahalli KU, Anseth KS (2010). Controlled two-photon photodegradation of PEG hydrogels to study and manipulate subcellular interactions on soft materials. Soft Matter.

[53] Kim J, Kong JS, Han W, Kim BS, Cho D-W (2020). 3D cell printing of tissue/organ-mimicking constructs for therapeutic and drug testing applications. IJMS.

[54] Ahadian, S. et al. Organ-on-a-chip platforms: a convergence of advanced materials, cells, and microscale technologies. *Adv. Healthcare Mater.*10.1002/adhm.201700506 (2017).

[55] Mittal R (2019). Organ‐on‐chip models: Implications in drug discovery and clinical applications. J. Cell Physiol..

[56] Van Norman GA (2020). Limitations of animal studies for predicting toxicity in clinical trials. JACC: Basic Transl. Sci..

[57] Gaetani R (2015). Epicardial application of cardiac progenitor cells in a 3D-printed gelatin/hyaluronic acid patch preserves cardiac function after myocardial infarction. Biomaterials.

[58] Norona LM, Nguyen DG, Gerber DA, Presnell SC, LeCluyse EL (2016). Editor’s highlight: modeling compound-induced fibrogenesis in vitro using three-dimensional bioprinted human liver tissues. Toxicol. Sci..

[59] Klein F (2011). Two-component polymer scaffolds for controlled three-dimensional cell culture. Adv. Mater..

[60] St. John, J. C. et al. in *Human Embryonic Stem Cell Protocols* Vol. 331 (ed Turksenvol, K) 347–374 (Humana Press, 2006).

[61] Prigione A, Fauler B, Lurz R, Lehrach H, Adjaye J (2010). The senescence-related Mitochondrial/Oxidative stress pathway is repressed in human induced pluripotent stem cells. Stem Cells.

[62] Wu J, Ocampo A, Belmonte JCI (2016). Cellular metabolism and induced pluripotency. Cell.

[63] Berger E (2018). Millifluidic culture improves human midbrain organoid vitality and differentiation. Lab Chip.

[64] Jiang BH, Semenza GL, Bauer C, Marti HH (1996). Hypoxia-inducible factor 1 levels vary exponentially over a physiologically relevant range of O2 tension. Am. J. Physiol.-Cell Physiol..

[65] Huang LE, Gu J, Schau M, Bunn HF (1998). Regulation of hypoxia-inducible factor 1 is mediated by an O2-dependent degradation domain via the ubiquitin-proteasome pathway. Proc. Natl Acad. Sci. USA.

[66] Greijer AE (2004). The role of hypoxia inducible factor 1 (HIF-1) in hypoxia induced apoptosis. J. Clin. Pathol..

[67] Wang M, Tan J, Miao Y, Li M, Zhang Q (2018). Role of Ca^2+^ and ion channels in the regulation of apoptosis under hypoxia. Histol. Histopathol..

[68] Punovuori K (2019). N-cadherin stabilises neural identity by dampening anti-neural signals. Development.

[69] Meng Q (2022). Human forebrain organoids reveal connections between valproic acid exposure and autism risk. Transl. Psychiatry.

[70] Qian X (2020). Sliced human cortical organoids for modeling distinct cortical layer formation. Cell Stem Cell.

[71] Qian X (2018). Generation of human brain region-specific organoids using a miniaturized spinning bioreactor. Nat. Protoc..

[72] Campbell K, Götz M (2002). Radial glia: multi-purpose cells for vertebrate brain development. Trends Neurosci..

[73] Barry DS, Pakan JMP, McDermott KW (2014). Radial glial cells: Key organisers in CNS development. Int. J. Biochem. Cell Biol..

[74] Saito T (2011). Neocortical layer formation of human developing brains and lissencephalies: consideration of layer-specific marker expression. Cereb. Cortex.

[75] Ozair MZ (2018). hPSC modeling reveals that fate selection of cortical deep projection neurons occurs in the subplate. Cell Stem Cell.

[76] Ip BK, Bayatti N, Howard NJ, Lindsay S, Clowry GJ (2011). The corticofugal neuron-associated genes ROBO1, SRGAP1, and CTIP2 exhibit an anterior to posterior gradient of expression in early fetal human neocortex development. Cereb. Cortex.

[77] Delgado RN (2022). Individual human cortical progenitors can produce excitatory and inhibitory neurons. Nature.

[78] Yu X, Zecevic N (2011). Dorsal radial glial cells have the potential to generate cortical interneurons in human but not in mouse brain. J. Neurosci..

[79] Tietz PS, Larusso NF (2006). Cholangiocyte biology. Curr. Opin. Gastroenterol..

[80] Ruebner BH, Blankenberg TA, Burrows DA, Soohoo W, Lund JK (1990). Development and transformation of the ductal plate in the developing human liver. Pediatr. Pathol..

[81] Limaye PB (2008). Expression of specific hepatocyte and cholangiocyte transcription factors in human liver disease and embryonic development. Lab Invest.

[82] Benet LZ, Sodhi JK (2022). Can in vitro*–i*n vivo extrapolation be successful? Recognizing the incorrect clearance assumptions. Clin. Pharma Therapeutics.

[83] Wang L (2015). How to choose in vitro systems to predict in vivo drug clearance: a system pharmacology perspective. BioMed. Res. Int..

[84] Di L (2012). A novel relay method for determining low-clearance values. Drug Metab. Dispos..

[85] Di L (2013). In vitro–in vivo correlation for low-clearance compounds using hepatocyte relay method. Drug Metab. Dispos..

[86] Murgasova R (2019). Further assessment of the relay hepatocyte assay for determination of intrinsic clearance of slowly metabolised compounds using radioactivity monitoring and LC–MS methods. Eur. J. Drug Metab. Pharmacokinet..

[87] Di L, Obach RS (2015). Addressing the challenges of low clearance in drug research. AAPS J..

[88] Dash A (2009). Liver tissue engineering in the evaluation of drug safety. Expert Opin. Drug Metab. Toxicol..

[89] McGinnity DF, Soars MG, Urbanowicz RA, Riley RJ (2004). Evaluation of fresh and cryopreserved hepatocytes as in vitro drug metabolism tools for the prediction of metabolic clearance. Drug Metab. Dispos..

[90] Godoy P (2013). Recent advances in 2D and 3D in vitro systems using primary hepatocytes, alternative hepatocyte sources and non-parenchymal liver cells and their use in investigating mechanisms of hepatotoxicity, cell signaling and ADME. Arch. Toxicol..

[91] Hutzler JM, Ring BJ, Anderson SR (2015). Low-turnover drug molecules: a current challenge for drug metabolism scientists. Drug Metab. Dispos..

[92] Lombardo F (2013). Comprehensive assessment of human pharmacokinetic prediction based on in vivo animal pharmacokinetic data, Part 2: clearance: the journal of clinical pharmacology. J. Clin. Pharm..

[93] Kurata Y, Muraki S, Hirota T, Araki H, Ieiri I (2021). Reduced theophylline clearance due to hepatic congestion secondary to right heart failure—a population pharmacokinetic study. Drug Metab. Pharmacokinetics.

[94] Ranga A (2016). Neural tube morphogenesis in synthetic 3D microenvironments. Proc. Natl Acad. Sci. USA.

[95] Medina JD (2020). Functionalization of alginate with extracellular matrix peptides enhances viability and function of encapsulated porcine islets. Adv. Healthc. Mater..

[96] Suong DNA (2021). Induction of inverted morphology in brain organoids by vertical-mixing bioreactors. Commun. Biol..

[97] Lancaster MA, Knoblich JA (2014). Generation of cerebral organoids from human pluripotent stem cells. Nat. Protoc..

[98] Boon R (2020). Amino acid levels determine metabolism and CYP450 function of hepatocytes and hepatoma cell lines. Nat. Commun..

[99] Roelandt, P., Vanhove, J. & Verfaillie, C. in *Pluripotent Stem Cells* (eds. Lakshmipathy, U. & Vemuri, M. C.) vol. 997 141–147 (Humana Press, 2013). 10.1007/978-1-62703-348-0_11 23546753

[100] Davies JE (2007). The pharmacological basis of therapeutics. Occup. Environ. Med..

[101] Stuart T (2019). Comprehensive integration of single-cell data. Cell Data. Cell.

[102] Cao J (2019). The single-cell transcriptional landscape of mammalian organogenesis. Nature.

[103] Kumar M (2021). A fully defined matrix to support a pluripotent stem cell derived multi-cell-liver steatohepatitis and fibrosis model. Biomaterials.

