# Supplementary material for: Large-scale perfused tissues via synthetic 3D soft microfluidics
Source: Nat Commun. 2023 Jan 12;14:193. doi: 10.1038/s41467-022-35619-1 (PMC9837048; doi:10.1038/s41467-022-35619-1)
Supplement: Supplementary file 1 — Supplementary Info [file 41467_2022_35619_MOESM1_ESM.pdf]

Large-scale perfused tissues via synthetic 3D soft microfluidics

Sergei Grebenyuk[1*], Abdel Rahman Abdel Fattah[1], Manoj Kumar[2], Burak Toprakhisar[2], Gregorius Rustandi[1], Anja Vananroye[3], Idris Salmon[1], Catherine Verfaillie[2], Mark Grillo[4], Adrian Ranga[1*]

[1] Laboratory of Bioengineering and Morphogenesis, Biomechanics Section, Department of Mechanical Engineering, KU Leuven, Leuven, Belgium
[2] Stem Cell Institute Leuven and Department of Development and Regeneration, Faculty of Medicine, KU Leuven, Leuven, Belgium
[3] Laboratory of Soft Matter, Rheology and Technology, Department of Chemical Engineering, KU Leuven, Leuven, Belgium
[4] Grillo Consulting Inc., San Francisco, California, United States

**email: sergii.grebeniuk@kuleuven.be, adrian.ranga@kuleuven.be*

Supplementary information

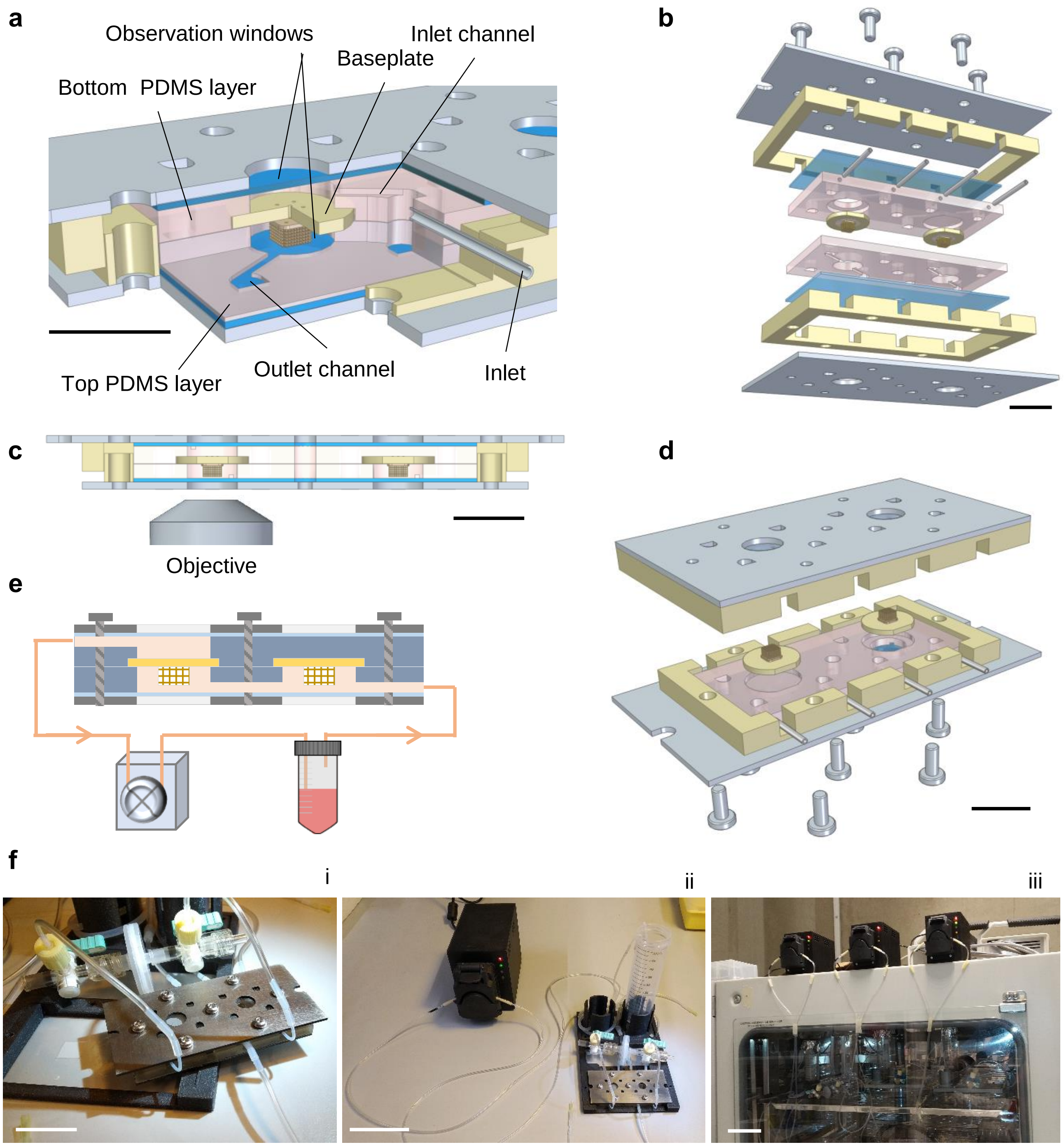

Supplementary Figure 1. Assembly and operation of the chip device. (**a**) Cutaway view of the fully assembled culture chip, the chamber with perfused capillary grid is shown. Cell culture medium enters the culture chamber through the inlet (1mm syringe needle, cut into 15mm pieces), passes through the capillary grid and leaves the chamber through outlet (not shown). Scale bar 10mm. (**b**) Exploded view of the chip. Scale bar 10mm. (**c**) Side view cross section of the chip. Tissue constructs can be observed through the

glass windows with inverted or upright microscopes. The view also demonstrates that the cell culture medium only comes into contact with PDMS(pink) and glass(blue) parts of the chip. Scale bar 10mm. (**d**) During the assembly, a user needs to handle only two pre-assembled “halves” of the chip. Scale bar 10mm. (**e**) Schematic representation of the perfusion system driven by a peristaltic pump, with culture medium circulating between the perfusion chip and the medium reservoir. Here, the grid in the right chamber is surrounded by a constant medium flow but the flow through its capillaries is absent, while in the downstream grid (left chamber) the flow passes through the capillaries and diffuses across the walls into the tissue. (**f**) Photographs of fully assembled chip and perfusion system; *Left*, the non-perfused and perfused chambers are connected by external tubing, with 0.22µm filter connected inline between the chambers. Scale bar 20mm. *Middle*, the chip and the medium reservoir resides in a custom 3D-printed holder. Scale bar 60mm. *Right*, the chip and the medium reservoir connected to a peristaltic pump installed outside of CO_2 incubator. Scale bar 60mm. The image shows three experiments running in parallel.

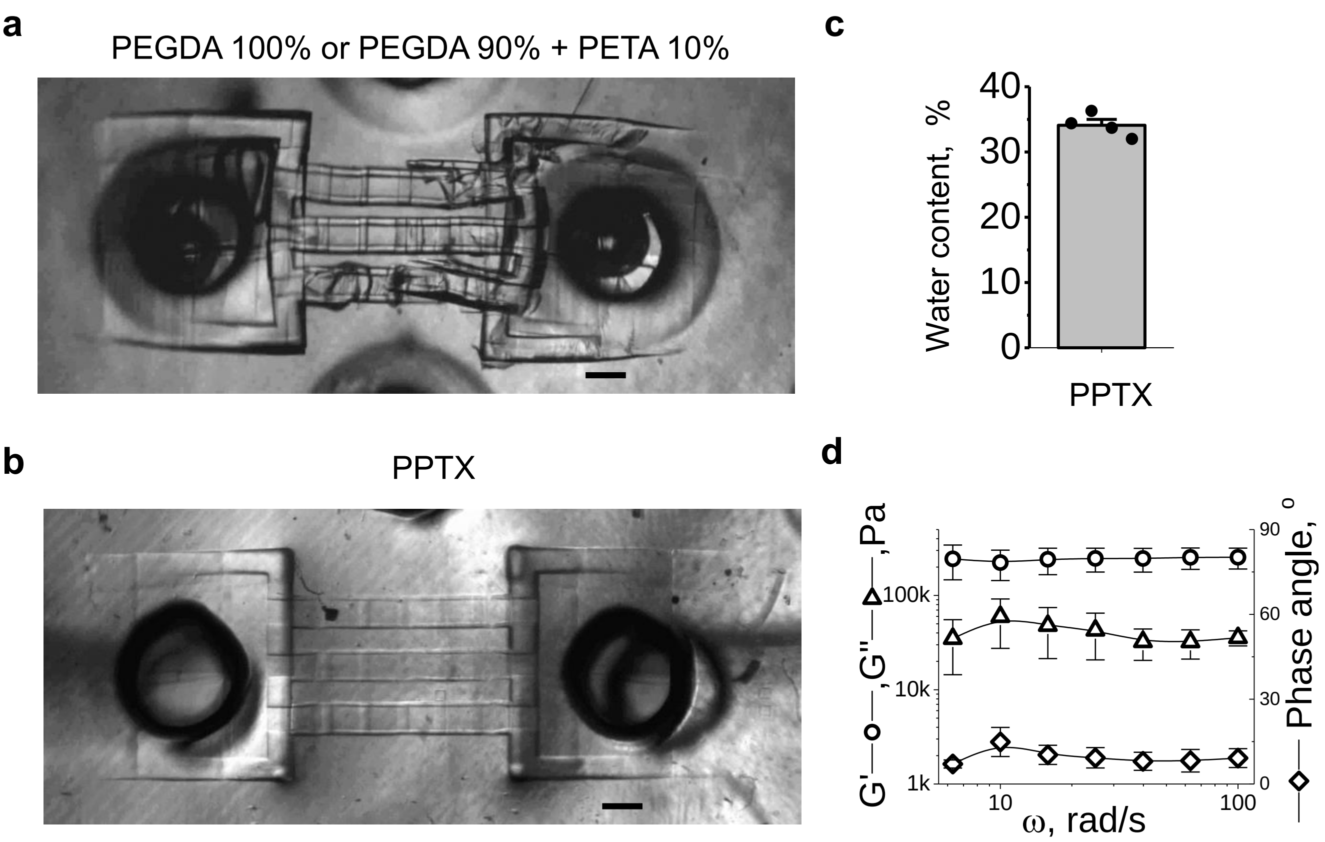

2

Supplementary Figure 2. Optimization and characterization of printable material properties. (**a**) Test structure printed from PEGDA 100% or PEGDA 90%/PETA 10% (PEGDA 90%) mixture. Both ends of the part are connected to a baseplate. When submerged to cell culture medium, swelling of the material leads to a breakage of the part (n=4 parts). Scale bar 100µm. (**b**) Our custom formulation (PPTX) resin effectively prevents swelling in aqueous media and preserves the geometry of the micro-fabricated structures (n=3 parts). Scale bar 100µm. (**c**) The mass percentage of water retained by PPTX photopolymer estimated at 34.1±0.8 % (n=4 samples). (d) Oscillatory shear stress tests (n=3 samples) estimated the storage modulus G' of PPTX polymer in the range of 250kPa, and the loss modulus in the range of 30kPa. Data are derived from independent experiments and represented as mean ± SEM

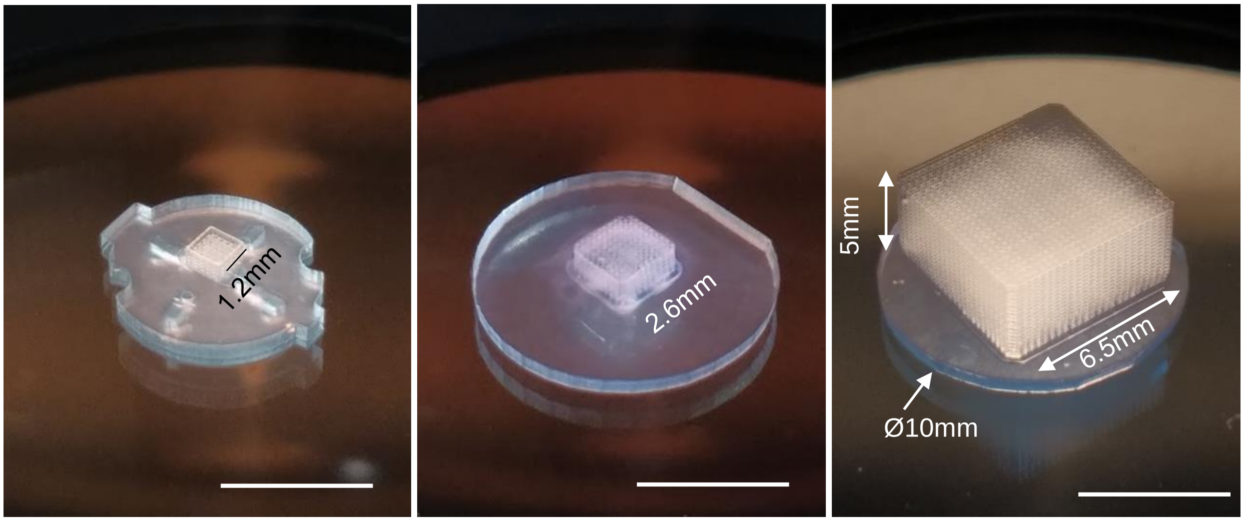

Supplementary Figure 3. Two-photon stereo-lithography enables precision fabrication in a wide range of scales. Microfluidic grids of different dimensions (in mm): 1.2mm x 1.2mm x 1.2mm (left, n>30 grids), 2.6mm x 2.6 mm x 1.5mm (middle, n>100 grids), 6.5mm x 6.5mm x 5mm (right, n=2 rgids). Perfusion vessels in each grid have identical 50µm diameter and inter-vessel distance of 250µm. Scale bar 5 mm. The printing time required to fabricate the parts was 40min, 3 hours and 17 hours respectively.

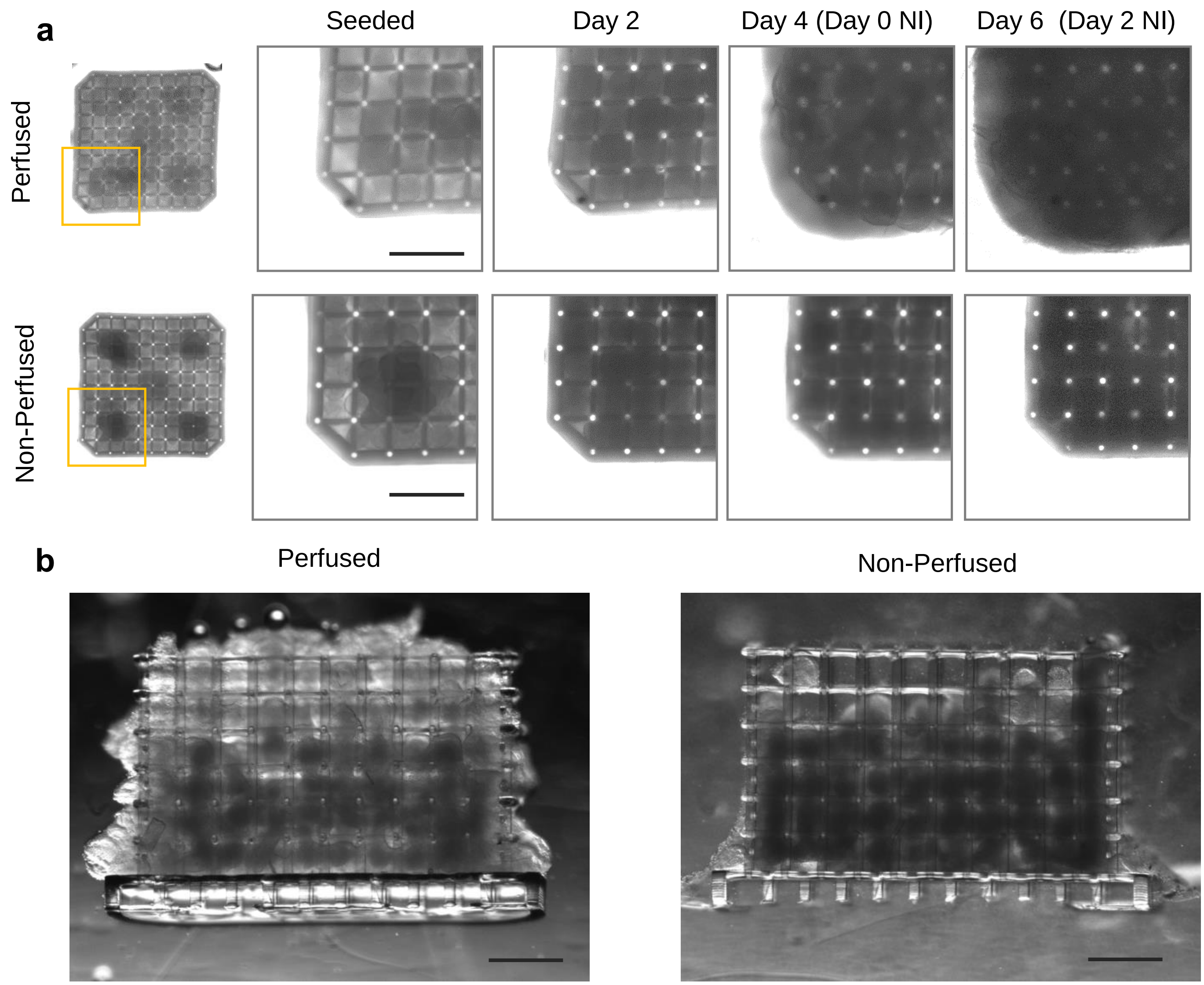

Supplementary Figure 4. Daily brightfield imaging of neural tissue in perfused and non-perfused chips. (**a**) Bright field images of the perfused (top) and non-perfused (bottom) tissue constructs taken every 2 days during the culturing protocol. (**b**) Live sections of perfused (left) and non-perfused (right) tissue constructs taken at the end of the culturing protocol (n=5 experiments). Scale bar 500µm.

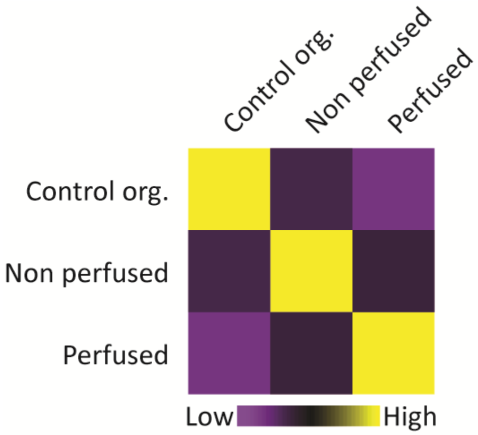

Supplementary Figure 5. Correlation analysis between perfused-, non-perfused neural tissue constructs and conventional neural organoid culture. Correlation analysis using the top 100 differentially expressed marker genes for each of the three experimental conditions.

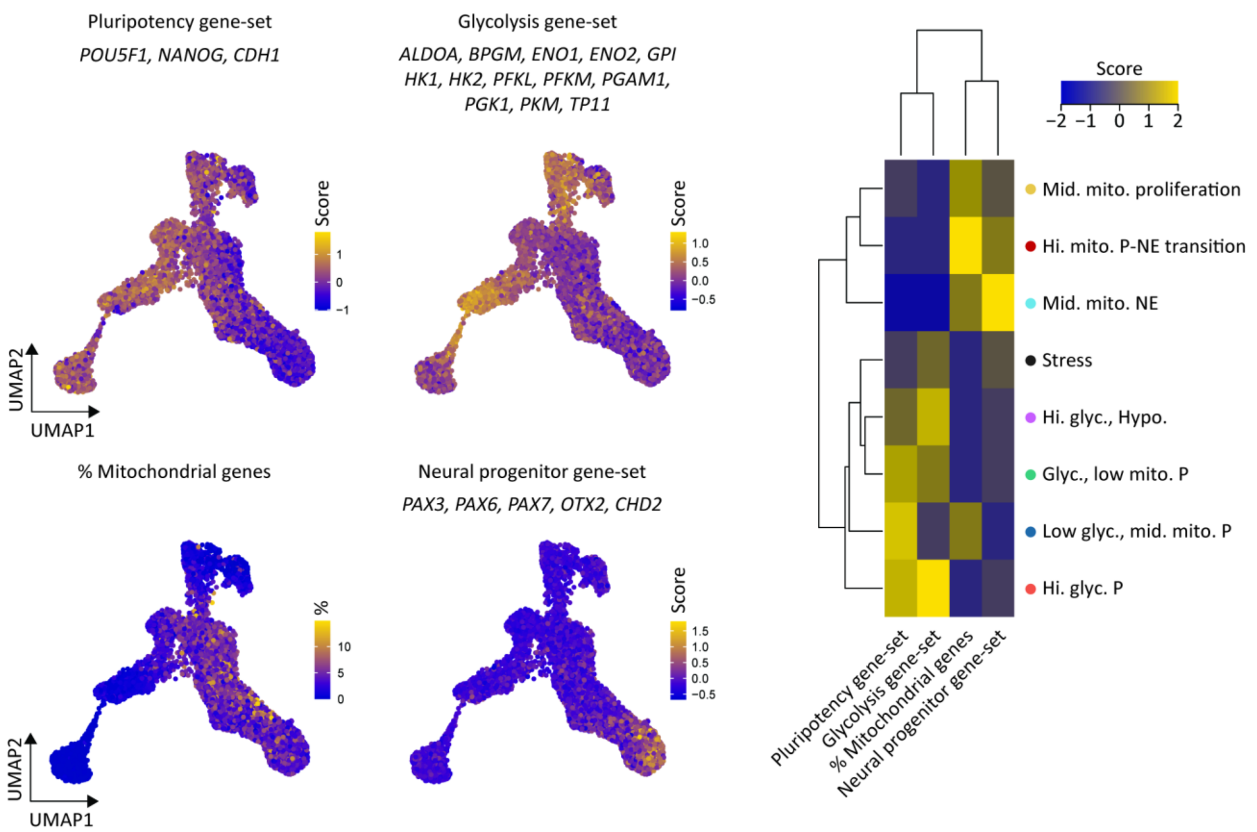

Supplementary Figure 6. Gene-set analysis for various processes. Combined dataset UMAP with scores for pluripotent, glycolysis and neural progenitor gene-set as well as the % mitochondrial genes. Hierarchical clustering of gene-set scores and % mitochondrial genes. Score is column scaled.

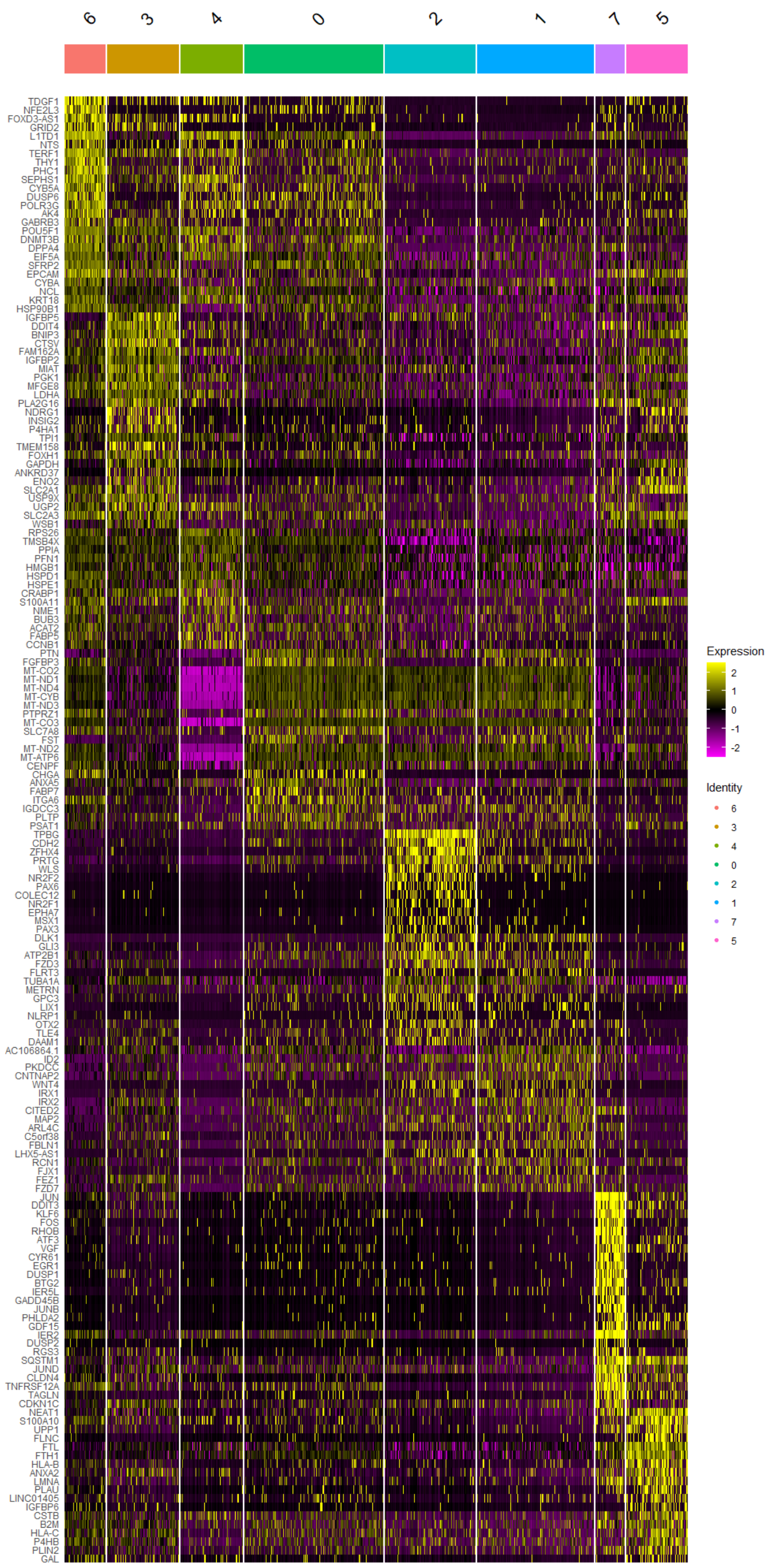

Supplementary Figure 7. Gene expression for unannotated hNTO clusters with the top 25 marker genes for each cluster. Color bar represents scaled gene expression.

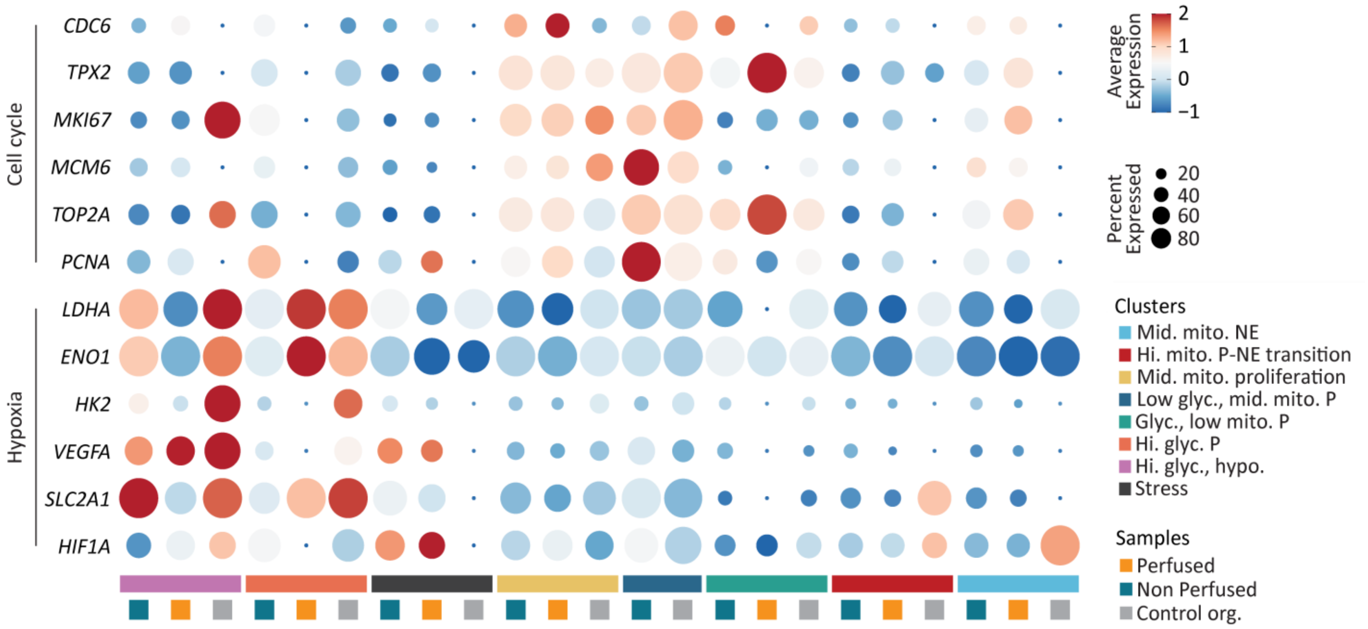

Supplementary Figure 8. Cluster-specific analysis of scRNAseq dataset. Dot-plot heatmap of hypoxia and cell cycle markers for each identified cluster. The average gene expression is represented by the color intensity of each dot, whereas the dot size represents the percentage of the gene-expressing cells for each sample within each cluster. Color bar represents scaled average gene expression.

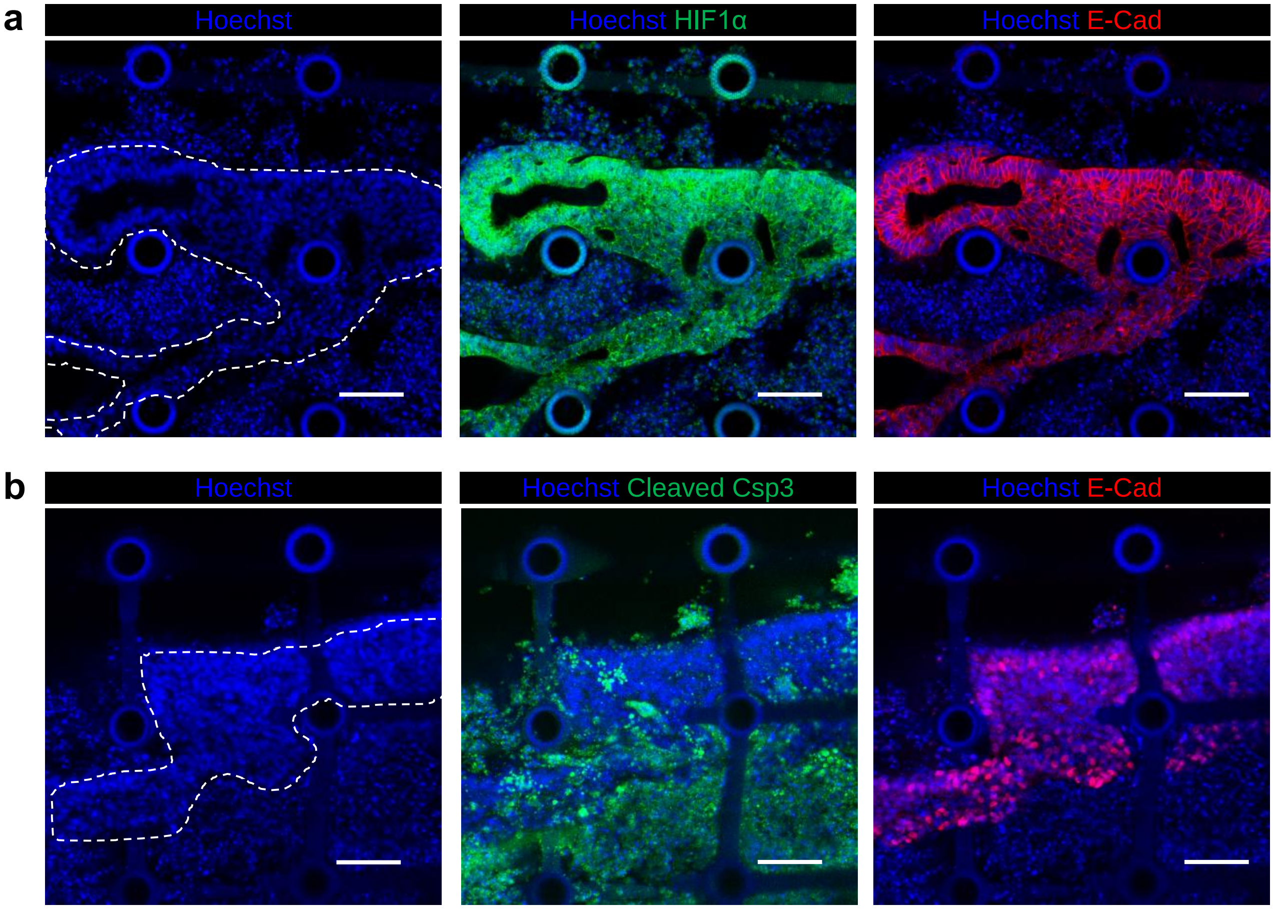

Supplementary Figure 9. Difference in localized expression of hypoxia marker HIF1α and apoptosis marker cleaved Caspase-3 after two days of neuronal induction in non-perfused grids. (**a**) HIF1α expression is localized to regions containing intact cell bodies (middle, n=3 independent experiments), evidenced by intact nuclei in the outlined region (left) as well as well-defined cytoplasmic regions stained with E-Cad antibody (right). (**b**) Cleaved Caspase-3 expression (middle) is localized to regions with apoptotic cell bodies (n=4 independent experiments) evidenced by Hoechst stain of fragmented nuclei (outside of the outlined live region on the left image). Scale bar 100µm.

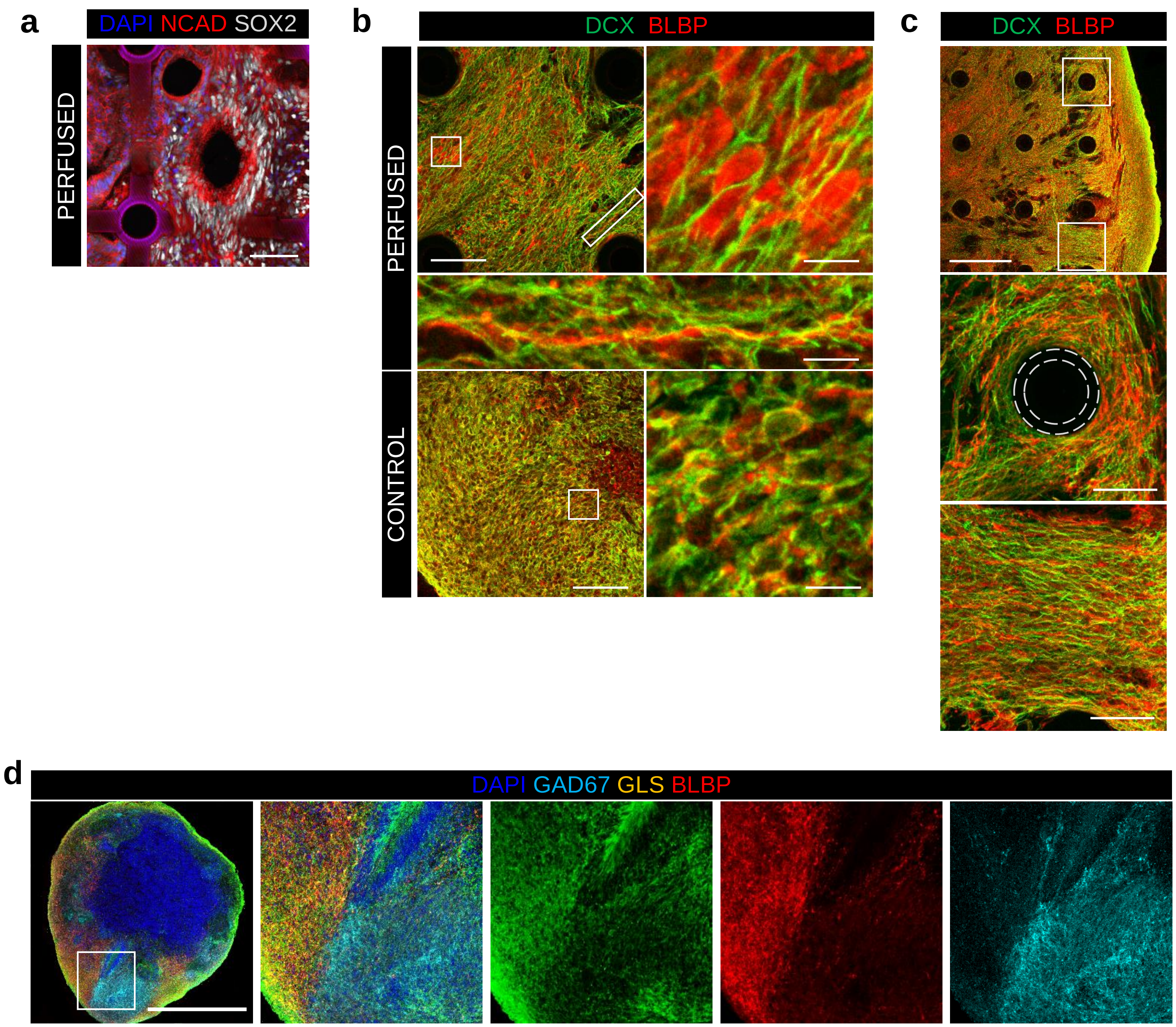

Supplementary Figure 10. Microarchitecture of organoids after long-term directed neural differentiation. (**a**) An example of a ventricle structure in perfused organoids. The inner wall of a SOX2+ ventricle is lined with N-Cadherin+ cells (red). Scale bar 70µm. (**b**) Perfused organoids develop extended neuronal (DCX+, green) and radial glia (BLBP+,red) processes. On the magnified images DCX+ processes align tightly with radial glia processes, while in control organoids the tissue, mainly lacking cellular outgrowths, retains a compact aspect, mainly composed of cellular bodies. Scale bars in the main and zoomed images are 70µm and 10µm respectively. (**c**) The alignment pattern of cellular processes in perfused organoids (top) ranges from circular (middle), where the tissue is adherent to the synthetic vessels, to parallel (bottom), where bundles of the processes bypass the vessels without encircling them.

Scale bars in the main and zoomed images are 250µm and 50µm respectively. (**d**) The images demonstrate segregated populations of GABAergic (GAD67, cyan) and glutamatergic (Glutaminase, green). Scale bars in the main and zoomed images are 500µm and 100µm respectively. Representative images were acquired in three independent experiments; all experiments produced similar results.

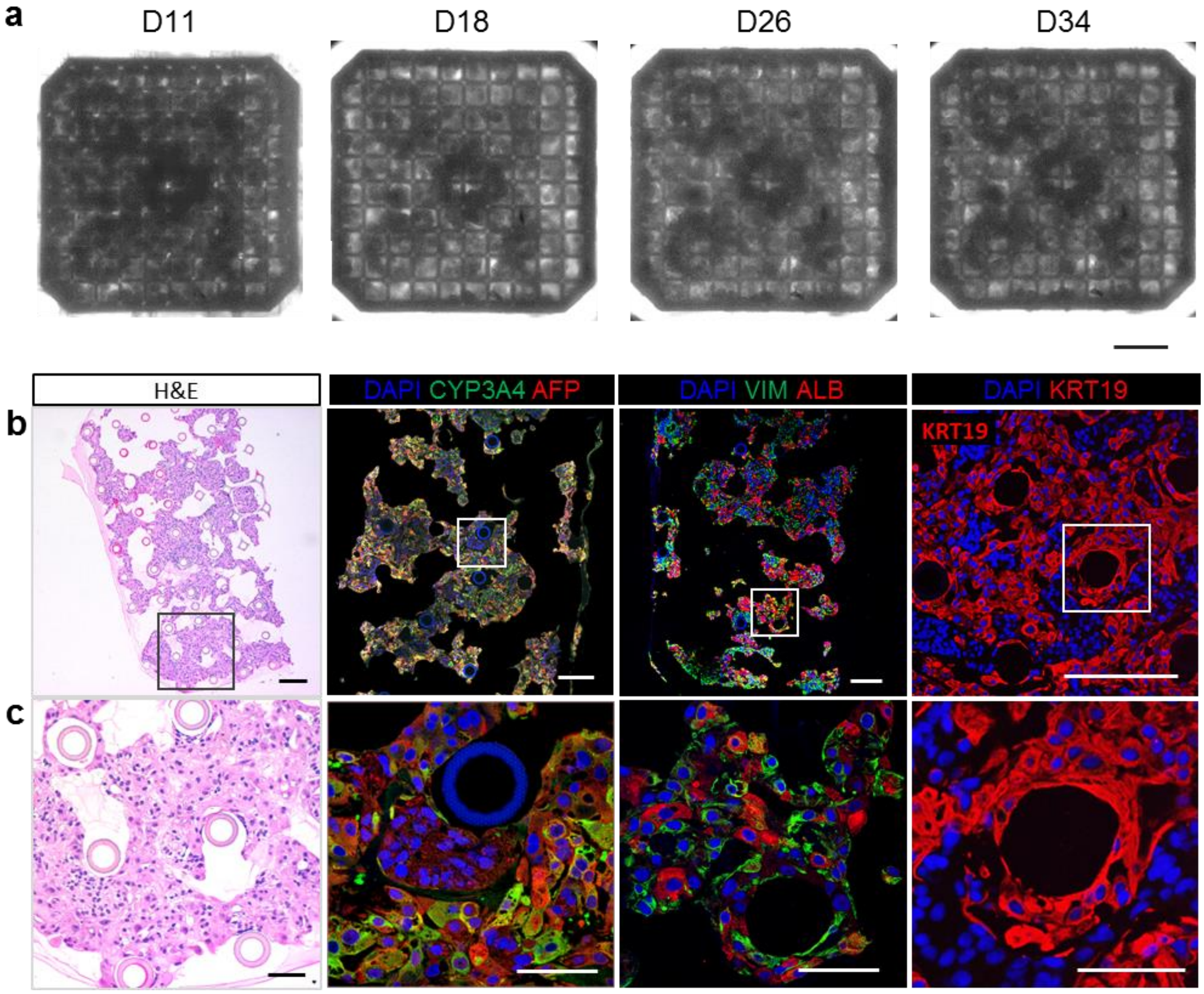

Supplementary Figure 11. Long term perfused liver tissue constructs. (**a**) Bright field images of the perfused liver-like tissue constructs taken during tissue culturing. Scale bar 500µm. (**b**) Immunostaining of 4µm paraffin sections, left to right: Eosin & Hematoxylin, CYP3A4(green) and Alpha Fetoprotein (red), Vimentin (green) and Albumin (red), Cytokeratin19 (red). Nuclear counterstain is shown in blue. Scale bar 200µm. Regions in squares are magnified and shown in (**c**) with scale bar 70 µm. Representative images were acquired in three independent experiments; all experiments produced similar results.

**Primary**	**Host**	**Dilution**	**Manufacturer**
Pax6, monoclonal[SC81649]	Mouse	1:200	Santa Cruz, sc-81649
Nanog	Goat	1:200	R&D Systems, AF1997
ECad, monoclonal [M168]	Mouse	1:500	Abcam, ab76055
NCad	Rabbit	1:200	Abcam, ab18203
Cleaved Caspase3	Rabbit	1:400	Cell Signaling Technology, 9661
HIF1a, monoclonal[EP1215Y]	Rabbit	1:500	Abcam, ab51608
HNF4α, monoclonal [K9218]	Mouse	1:200	Abcam, ab41898
Alpha-1-Antitrypsin	Rabbit	1:200	DAKO, A0012 (00092029)
PEPCK, monoclonal[E-1]	Mouse	1/1000	Santa Cruz, sc-271204
MRP2, monoclonal [M2 III-6]	Mouse	1/500	Abcam, ab3373
Cytokeratin 19	goat	1/500	Santa Cruz, sc-33120
ALB, monoclonal [EPR20195]	Rabbit	1/500	Abcam, ab207327
BLBP	Goat	1/250	Abcam, ab110099
GAD67, monoclonal[K-87]	Mouse	1/100	Abcam, ab26116
Glutaminase, monoclonal[EP7212]	Rabbit	1/250	Abcam, ab156876
SATB2, monoclonal [SATBA4B10]	Mouse	1/250	Abcam, ab51502
CTIP2, monoclonal[25B6]	Rat	1/250	Abcam, ab18465
TBR1	Rabbit	1/250	Abcam, ab31940
MBP, monoclonal[SMI99]	Mouse	1/250	Biolegend, 808401
NeuN, monoclonal[A60]	Mouse	1/300	Merck (MAB377)
SOX2	Goat	1/100	R&D systems (AF2018)
**Secondary**			
Hoechst			Sigma-Aldrich, 14533
Anti-mouse Alexa 647	Donkey	1:500	Invitrogen, A31571
Anti-goat Alexa 647	Donkey	1:500	Invitrogen, A21447
Anti-rat Alexa 555	Goat	1:500	Invitrogen, A21434
Anti-goat Alexa 555	Donkey	1:500	Invitrogen, A21432
Anti-rabbit Alexa 555	Donkey	1:500	Invitrogen, A31572
Anti-mouse Alexa 488	Donky	1:500	Invitrogen, A11029

Supplementary Table 1. List of antibodies used in this study

RPL19	ATTGGTCTCATTGGGGTCTAAC<br>AGTATGCTCAGGCTTCAGAAGA
AAT	AGGGCCTGAAGCTAGTGGAT<br>TCCTCGGTGTCCTTGACTTC
NTCP	ATGCTGAGGCAAAGGATGTC<br>AGCAGCAGCACGACAGAGTA
G6PC	GTGTCCGTGATCGCAGACC<br>GACGAGGTTGAGCCAGTCTC
CYP3A4	TTCCTCCCTGAAAGATTCAGC<br>GTTGAAGAAGTCCTCCTAAGCT
PEPCK	AAGAAGTGCTTTGCTCTCAG<br>CCTTAAATGACCTTGTGCGT
CYP2D6	CATACCTGCCTCACTACCAAA<br>TGTCCTGCCTGGTCCTC
PGC1α	CCTTGCAGCACAAGAAAACA<br>TGCTTCGTCGTCAAAAACAG
HNF6	AAATCACCATTTCCCAGCAG<br>ACTCCTCCTTCTTGCGTTCA
ALB	ATGCTGAGGCAAAGGATGTC<br>AGCAGCAGCACGACAGAGTA
AAT	AGGGCCTGAAGCTAGTGGAT<br>TCCTCGGTGTCCTTGACTTC

Supplementary Table 2. List of primers used in this study
