# Supplementary material for: Large-scale perfused tissues via synthetic 3D soft microfluidics
Source: Nat Commun. 2023 Jan 12;14:193. doi: 10.1038/s41467-022-35619-1 (PMC9837048; doi:10.1038/s41467-022-35619-1)
Supplement: Supplementary file 2 — Reporting Summary [file 41467_2022_35619_MOESM2_ESM.pdf]

nature portfolio

Corresponding author(s): Adrian Ranga, Sergei Grebenyuk

Last updated by author(s): 29/11/2022

Reporting Summary

Nature Portfolio wishes to improve the reproducibility of the work that we publish. This form provides structure for consistency and transparency in reporting. For further information on Nature Portfolio policies, see our Editorial Policies and the Editorial Policy Checklist.

Statistics

For all statistical analyses, confirm that the following items are present in the figure legend, table legend, main text, or Methods section.

n/a	Confirmed	
☐	☒	The exact sample size (*n*) for each experimental group/condition, given as a discrete number and unit of measurement
☐	☒	A statement on whether measurements were taken from distinct samples or whether the same sample was measured repeatedly
☐	☒	The statistical test(s) used AND whether they are one- or two-sided<br>*Only common tests should be described solely by name; describe more complex techniques in the Methods section.*
☒	☐	A description of all covariates tested
☒	☐	A description of any assumptions or corrections, such as tests of normality and adjustment for multiple comparisons
☐	☒	A full description of the statistical parameters including central tendency (e.g. means) or other basic estimates (e.g. regression coefficient) AND variation (e.g. standard deviation) or associated estimates of uncertainty (e.g. confidence intervals)
☐	☒	For null hypothesis testing, the test statistic (e.g. *F*, *t*, *r*) with confidence intervals, effect sizes, degrees of freedom and *P* value noted<br>*Give P values as exact values whenever suitable.*
☒	☐	For Bayesian analysis, information on the choice of priors and Markov chain Monte Carlo settings
☒	☐	For hierarchical and complex designs, identification of the appropriate level for tests and full reporting of outcomes
☐	☒	Estimates of effect sizes (e.g. Cohen's *d*, Pearson's *r*), indicating how they were calculated

Our web collection on statistics for biologists contains articles on many of the points above.

Software and code

Policy information about availability of computer code

Data collection	Image acquisition was performed using Leica LAS v.X 5.1.0
Data analysis	GraphPad Prism v.9.4, OriginLab Origin Pro v9.5, R Studio v.3.5.2, Seurat R Package v3.0.1, ImageJ v.1.52, 1.53.

For manuscripts utilizing custom algorithms or software that are central to the research but not yet described in published literature, software must be made available to editors and reviewers. We strongly encourage code deposition in a community repository (e.g. GitHub). See the Nature Portfolio guidelines for submitting code & software for further information.

Data

Policy information about availability of data

All manuscripts must include a data availability statement. This statement should provide the following information, where applicable:

- Accession codes, unique identifiers, or web links for publicly available datasets
- A description of any restrictions on data availability
- For clinical datasets or third party data, please ensure that the statement adheres to our policy

All raw sequencing data, and the combined processed and metadata files generated in this study are available at GEO. The accession number for the reported data is GSE181290 (https://www.ncbi.nlm.nih.gov/geo/query/acc.cgi?acc=GSE181290). This study did not generate any unique code.

Field-specific reporting

Please select the one below that is the best fit for your research. If you are not sure, read the appropriate sections before making your selection.

☒ Life sciences ☐ Behavioural & social sciences ☐ Ecological, evolutionary & environmental sciences

For a reference copy of the document with all sections, see nature.com/documents/nr-reporting-summary-flat.pdf

Life sciences study design

All studies must disclose on these points even when the disclosure is negative.

Sample size	Experiments were conducted with a minimum sample size of n=3 independent biological replicates (up to n=12 independent biological replicates), with several technical replicates depending on the experiment (except for scRNAseq data). Multiple field of views were quantified for each biological replicates were used for quantification. Sample size was selected following common practice in the organoid field (Fattah et al. Nat. Comm. 2021, Boon et al. Nat. Comm. 2020; Drakhlis et al. Nat. Biotech. 2021; Lewis-Israeli et al. Nature 2021; Branco et al. Nat. Comm. 2022).
Data exclusions	All available IHC samples were included in analysis. Cells in the scRNAseq analysis were excluded from the analysis when their level of genes rising from the mitochondrial genome exceeded 15%. Additionally cells with fewer than 200 (low quality) and more than 7,500 (potential doublets) detected genes were also filtered out.
Replication	Data was replicated using a minimum of 3 and a maximum of 12 independent experiments to ensure results were reproducible. Number of independent biological replications are given in figure legends. Organoids and tissues were obtained from several rounds of differentiation and using two differentiation protocols to ensure reproducibility.
Randomization	The starting and culture conditions were kept constant to eliminate sample-specific characteristics, leaving the effect of perfusion and effect of particular tissue generation method (organoids vs tissue constructs) responsible for the observed variations. Due to the nature of the experimental setting, no randomization was possible, as the control organoids, perfused and non-perfused tissue samples were obtained as the result of a specific experimental condition.
Blinding	The study was not blinded. Quantifications were performed using computational pipeline applied equally to all conditions and replicates for a given IHC marker in order to limit investigator bias.

Reporting for specific materials, systems and methods

We require information from authors about some types of materials, experimental systems and methods used in many studies. Here, indicate whether each material, system or method listed is relevant to your study. If you are not sure if a list item applies to your research, read the appropriate section before selecting a response.

Materials & experimental systems

n/a	Involved in the study	
☐	☒	Antibodies
☐	☒	Eukaryotic cell lines
☒	☐	Palaeontology and archaeology
☒	☐	Animals and other organisms
☒	☐	Human research participants
☒	☐	Clinical data
☒	☐	Dual use research of concern

Methods

n/a	Involved in the study	
☒	☐	ChIP-seq
☒	☐	Flow cytometry
☒	☐	MRI-based neuroimaging

Antibodies

Antibodies used

Anti-Pax6, mouse monoclonal[SC81649] IgG1, Santa Cruz (sc-81649), dilution 1:250
Anti-Nanog, goat polyclonal IgG, R&D Systems (AF1997), dilution 1:200
Anti-E Cadherin mouse monoclonal [M168] IgG1, Abcam (ab76055), dilution 1:500
Anti-N Cadherin rabbit polyclonal IgG, Abcam (ab18203), dilution, 1:500
Anti-Cleaved Caspase3 rabbit polyclonal IgG Cell Signaling Technology (9661), dilution 1:400
Anti-HIF1a, Rabbit monoclonal[EP1215Y] Abcam IgG2a (ab51608), dilution 1:500
Anti-HNF4α, mouse monoclonal [K9218] Abcam (ab41898), dilution 1:200
Anti-Alpha-1-Antitrypsin, rabbit polyclonal DAKO (A0012), dilution 1:200
Anti-PEPCK mouse monoclonal[E-1] IgG2b, Santa Cruz (sc-271204), dilution 1:1000
Anti-MRP2, mouse monoclonal [M2 III-6] iGg2A, Abcam (ab3373), dilution 1:500
Anti-Cytokeratin 19, goat polyclonal, Santa Cruz (sc-33120), dilution, 1:500
Anti-Albumin rabbit monoclonal [EPR20195], Abcam (ab207327), dillution 1:500

Anti-BLBP goat polyclonal IgG, Abcam (ab110099)
Anti-GAD67 mouse monoclonal[K-87] IgG1, Abcam (ab26116)
Anti-Glutaminase rabbit monoclonal[EP7212] Abcam (ab156876)
Anti-SATB2 mouse monoclonal [SATBA4B10] IgG1 Abcam (ab51502)
Anti-CTIP2 rat monoclonal[25B6] IgG2a, Abcam (ab18465)
Anti-TBR1 rabbit polyclonal IgG, Abcam (ab31940)
Anti-Myelin Basic Protein mouse monoclonal[SMI99] IgG2b,k Biolegend(808401), previously Covance (SMI-99P)
Anti-NeuN mouse monoclonal[A60] IgG1, Merck (MAB377)
Anti-SOX2 goat polyclonal IgG, R&D systems (AF2018)

Validation

The following antibodies were used in this study. All antibodies were validated in multiple research reports, selected references are provided for each antibody. Only certified and company-validated antibodies were used for IHC. Cellular localization of all investigated proteins have previously been reported and match the reported expressions in this study.

Anti-Pax6, mouse monoclonal[SC81649] IgG1, Santa Cruz (sc-81649), dilution 1:250
Cited in 39 publications. Recent relevant citations (PMID) – 36123835, 35944314, 35394834, 36403550, 36161772, 35085946, 35910668, 35231638

Anti-Nanog, goat polyclonal IgG, R&D Systems (AF1997), dilution 1:200
Cited in 190 publications. Recent relevant citations (PMID) – 35974013, 35659878, 34130154, 34004179, 31677955, 31204176, 30244870

Anti-E Cadherin mouse monoclonal [M168] IgG1, Abcam (ab76055), dilution 1:500
Cited in 312 publications. Recent relevant citations (PMID) – 31699989, 30936544, 30931940, 30405106, 30467425, 34023817, 33627414

Anti-N Cadherin rabbit polyclonal IgG, Abcam (ab18203), dilution, 1:500
Cited in 620 publications. Recent relevant citations (PMID) – 33723434, 33664246, 33171523, 34429761, 33762953, 35778954, 35902569

Anti-Cleaved Caspase3 rabbit polyclonal IgG Cell Signaling Technology (9661), dilution 1:400
Cited in 1000+ publications. Recent relevant citations (PMID) – 30228264, 29335016, 29142217, 34071893, 33259798, 30146491, 29973717

Anti-HIF1a, Rabbit monoclonal[EP1215Y] Abcam IgG2a (ab51608), dilution 1:500
Cited in 244 publications. Recent relevant citations (PMID) – 33888696, 33921614, 30833558, 30962421, 30952842, 30479336, 29180628

Anti-HNF4α, mouse monoclonal [K9218] Abcam (ab41898), dilution 1:200
Cited in 83 publications. Recent relevant citations (PMID) – 32770044, 33052062, 34505013, 33810128, 32764140, 32051143, 32747424

Anti-Alpha-1-Antitrypsin, rabbit polyclonal DAKO (A0012), dilution 1:200
Cited in 52 publications. Recent relevant citations (PMID) – 34788088, 35211754, 34276045, 33671632, 33545341

Anti-PEPCK mouse monoclonal[E-1] IgG2b, Santa Cruz (sc-271204), dilution 1:1000
Cited in 4 publications. Recent relevant citations (PMID) - 29874126, 28710665, 26782298, 26445495

Anti-MRP2, mouse monoclonal (M2 III-6) iGg2A, Abcam (ab3373), dilution 1:500
Cited in 72 publications. Recent relevant citations (PMID) – 33953166, 30664215, 33216752, 32814794, 31904483, 32571333, 29464551

Anti-Cytokeratin 19, goat polyclonal, Santa Cruz (sc-33120), dilution, 1:500
Recent relevant citations (PMID) – 18511743, 19234059

Anti-Albumin rabbit monoclonal [EPR20195], Abcam (ab207327), dillution 1:500
Cited in 39 publications. Recent relevant citations (PMID) – 33397952, 33708336, 34022892, 34026460, 33922642, 34459124 31992692

Anti-BLBP goat polyclonal IgG, Abcam (ab110099)
Relevant citation (PMID) – 31850342, 34428340. Tested by manufacturer in IHC applications in human tissues.

Anti-GAD67 mouse monoclonal[K-87] IgG1, Abcam (ab26116)
Cited in 75 publications. Recent relevant citations (PMID) – 32203578, 33636387, 32507685, 32174013, 32334536, 32341334, 31481756

Anti-Glutaminase rabbit monoclonal[EP7212] Abcam (ab156876)
Knockout validated by manufacturer. Cited in 60 publications. Recent relevant citations (PMID) – 33596851, 33649795, 33854374, 33947068, 32134147, 31866442, 33004309

Anti-SATB2 mouse monoclonal [SATBA4B10] IgG1 Abcam (ab51502)
Cited in 206 publications. Recent relevant citations (PMID) – 33431859, 33723434, 33708771, 33523829, 33558651, 34426698, 31901251

Anti-CTIP2 rat monoclonal[25B6] IgG2a, Abcam (ab18465)
Cited in 567 publications. Recent relevant citations (PMID) – 32051547, 32995858, 33723434, 33765444, 33789888, 33891871, 33976153

Anti-TBR1 rabbit polyclonal IgG, Abcam (ab31940)
Cited in 342 publications. Recent relevant citations (PMID) – 33433624, 33501759, 33510137, 33789767, 33789888, 33891871, 33893219

Anti-Myelin Basic Protein mouse monoclonal[SMI99] IgG2b,k Biolegend(808401), previously Covance (SMI-99P)
Cited in 43 publications. Recent relevant citations (PMID) – 29348594, 29937715, 29596047, 30094607, 29997299, 30449600, 30559305

Anti-NeuN mouse monoclonal[A60] IgG1, Merck (MAB377)
Validated by manufacturer for IF/IHC/ICC in human. Recent relevant citations (PMID) – 24723034, 24911405, 24748560, 26203299, 25834048, 25834052, 25870539

Anti-SOX2 goat polyclonal IgG, R&D systems (AF2018)
Cited in 173 publications. Recent relevant citations (PMID) – 36001974, 32294450, 35355018, 35496911, 33887179, 34244682, 33838105

Eukaryotic cell lines

Policy information about cell lines

Cell line source(s)	Sigma 0028, Sigma-Aldrich<br>hIPS cell line for liver differentiation reported in Boon, R. et al. Nat Commun 11, 1393 (2020)
Authentication	All hIPSC cell lines have been authenticated by the original sources or internally by immunostaining for pluripotency markers
Mycoplasma contamination	All cell lines tested negative for mycoplasma contamination
Commonly misidentified lines<br>(See ICLAC register)	No commonly misidentified cell lines listed by ICLAC were used in this work
